# A four-week minimalist shoe walking intervention influences foot posture and balance in young adults–a randomized controlled trial

**DOI:** 10.1371/journal.pone.0304640

**Published:** 2024-06-20

**Authors:** Anna Gabriel, Katharina Fuchs, Bernhard Haller, Iwona Sulowska-Daszyk, Thomas Horstmann, Andreas Konrad

**Affiliations:** 1 Professorship for Conservative and Rehabilitative Orthopedics, School of Medicine and Health, Technical University of Munich, Munich, Germany; 2 Institute of AI and Informatics in Medicine, School of Medicine and Health, University Hospital Klinikum rechts der Isar, Technical University of Munich, Munich, Germany; 3 Institute of Clinical Rehabilitation, University of Physical Education in Kraków, Kraków, Poland; 4 Institute of Human Movement Science, Sport and Health, Graz University, Graz, Austria; UFPE: Universidade Federal de Pernambuco, BRAZIL

## Abstract

**Introduction:**

Minimalist shoes (MS) are beneficial for foot health. The foot is a part of the posterior chain. It is suggested that interventions on the plantar foot sole also affect the upper segments of the body. This study aimed to investigate the local and remote effects along the posterior chain of four weeks of MS walking in recreationally active young adults.

**Methods:**

28 healthy participants (15 female, 13 male; 25.3 ± 5.3 years; 70.2 ± 11.9 kg; 175.0 ± 7.8 cm) were randomly assigned to a control- or intervention group. The intervention group undertook a four-week incremental MS walking program, which included 3,000 steps/day in the first week, increasing to 5,000 steps/day for the remaining three weeks. The control group walked in their preferred shoe (no MS). We assessed the following parameters in a laboratory at baseline [M1], after the four-week intervention [M2], and after a four-week wash-out period [M3]: Foot parameters (i.e., Foot Posture Index-6, Arch Rigidity Index), static single-leg stance balance, foot-, ankle-, and posterior chain range of motion, and muscle strength of the posterior chain. We fitted multiple hierarchically built mixed models to the data.

**Results:**

In the MS group, the Foot Posture Index (b = -3.72, t(51) = -6.05, p < .001, [-4.94, 2.51]) and balance (b = -17.96, t(49) = -2.56, p = .01, [-31.54, 4.37]) significantly improved from M1 to M2, but not all other parameters (all p >.05). The improvements remained at M3 (Foot Posture Index: b = -1.71, t(51) = -2.73, p = .009, [-4,94,0.48]; balance: b = -15.97, t(49) = -2.25, p = .03, [-29.72, 2.21]).

**Discussion:**

Walking in MS for four weeks might be advantageous for foot health of recreationally active young adults but no chronic remote effects should be expected.

## Introduction

With 26 bones, 32 joints, over 100 ligaments, and further soft tissue and muscular structures, the human foot provides a mobile and stable basis during movement in an upright position [1]. Authors of prior studies raised concerns that habitual, cushioned footwear, shoe inserts, and orthotics, which often do not support the natural shape of the foot and impair flexibility, might lead to anatomical and functional changes and contribute to the weakness of the intrinsic foot muscles (IFM) [2–6], which is associated with orthopedic foot diseases (e.g., hallux valgus) [4, 7, 8]. Populations wearing minimalist shoes (MS), which are characterized by a light weight, flexible sole, no cushioning, and no motion control [9–11], show differences in foot statics, e.g., wider feet [12] and a decreased hallux angle [13, 14].

Moreover, IFM weakness causes changes in foot posture towards a pronated position, which may cause dysfunction or overloading of the knee joint and upper parts of the kinematic chain [15, 16]. IFM strength and size, mainly of the abductor hallucis, abductor digiti minimi, and flexor digitorum brevis muscles [3, 17–19], play a key role in the function of the medial longitudinal arch (MLA) during weight bearing [3, 4, 7, 8]. During walking, the MLA absorbs impact forces and helps to maintain midtarsal rigidity for powered plantar flexion during toe-off [20]. Thus, IFM provide both foot stability and flexibility for shock absorption [21]. Foot exercises are traditionally recommended as therapy to improve IFM strength and foot health [22, 23]. Wearing MS was shown to be similarly effective in increasing the size and strength of the IFM [23], which leads to improved support of the MLA and better control of foot pronation [24].

Wearing MS also seems to positively correlate with balance skills, associated with IFM functioning, and consequently lowers the risk of falls [4, 8, 9, 19, 25]. MS might also stimulate the plantar receptors of the foot, ankle joint, and musculotendinous receptors [26], which may improve sensorimotor function [26–29]. At the same time, somatosensory feedback is reduced in standard shoes due to the cushioning protective layer between the ground and the foot [30]. A further aspect of MS is that the greater flexibility of the midsole—compared to conventional shoes—allows the foot to go through a greater range of motion (ROM) during the stance phase of walking and promotes greater ankle dorsiflexion before ground contact [19, 31, 32].

Running in MS was reported to not only have local effects but also acutely increases remote posterior chain (PC) muscle activity, namely of the calf- and gluteal muscles, during running [33]. The PC comprises the structures of the plantar surface to the dorsal lower extremity and back linked via connective tissue [34]. Azevedo, Mezêncio [35] also found higher remote biceps femoris muscle activity during running after 16 weeks of MS running compared to habitual running shoes. Moreover, foam rolling or stretching interventions on the plantar surface can acutely and chronically increase remote PC ROM [36–38]. Although there is evidence concerning remote PC effects of running with MS and plantar surface treatment, not much is known about the chronic remote effects of MS walking on various parameters of the PC.

Although prior MS studies were mainly performed on runners, the recreationally active, young population might also benefit from MS [9, 39]. Different from prior studies, we assessed healthy, recreationally active young adults (i.e., non-runners). Further, there is little evidence on the chronic local and remote effects on the PC after a four-week MS walking intervention and a four-week wash-out period.

### Specific objectives and hypotheses

Therefore, this study aimed to (1) investigate the effects of a four-week MS walking intervention on foot posture, MLA rigidity, single-leg stance balance, and foot and ankle ROM. In addition, we also aimed to investigate the (2) remote ROM and strength effects along the PC and (3) assessed whether potential effects remained after a four-week post-intervention wash-out phase. We hypothesized that similar to prior studies on running, due to increased IFM strength, improvements in foot posture, MLA rigidity, and balance improvements can be found in a healthy, young population after a four-week MS walking intervention. Further, we assume that walking in MS could influence foot and ankle ROM as the flexible sole allows the foot to go through a greater ROM. As indicated by prior studies, we expect remote effects along the PC (i.e., strength and ROM).

## Materials and methods

This study was conducted in accordance with the ethical principles of the Declaration of Helsinki as part of a larger study, which is registered in the German Clinical Trial Register (DRKS00027923, 24/08/2022) and approved by the ethics committee of the Technical University of Munich (2022-114-S-KK). We applied for study registration on 8 April 2022. After all required revisions were performed and the ethics approval was obtained and uploaded, the study was registered on 24 August 2022. Participants provided written informed consent before the study. The study followed the CONSORT guideline for randomized controlled trials.

### Trial design, procedures, and settings

**Fig 1:**
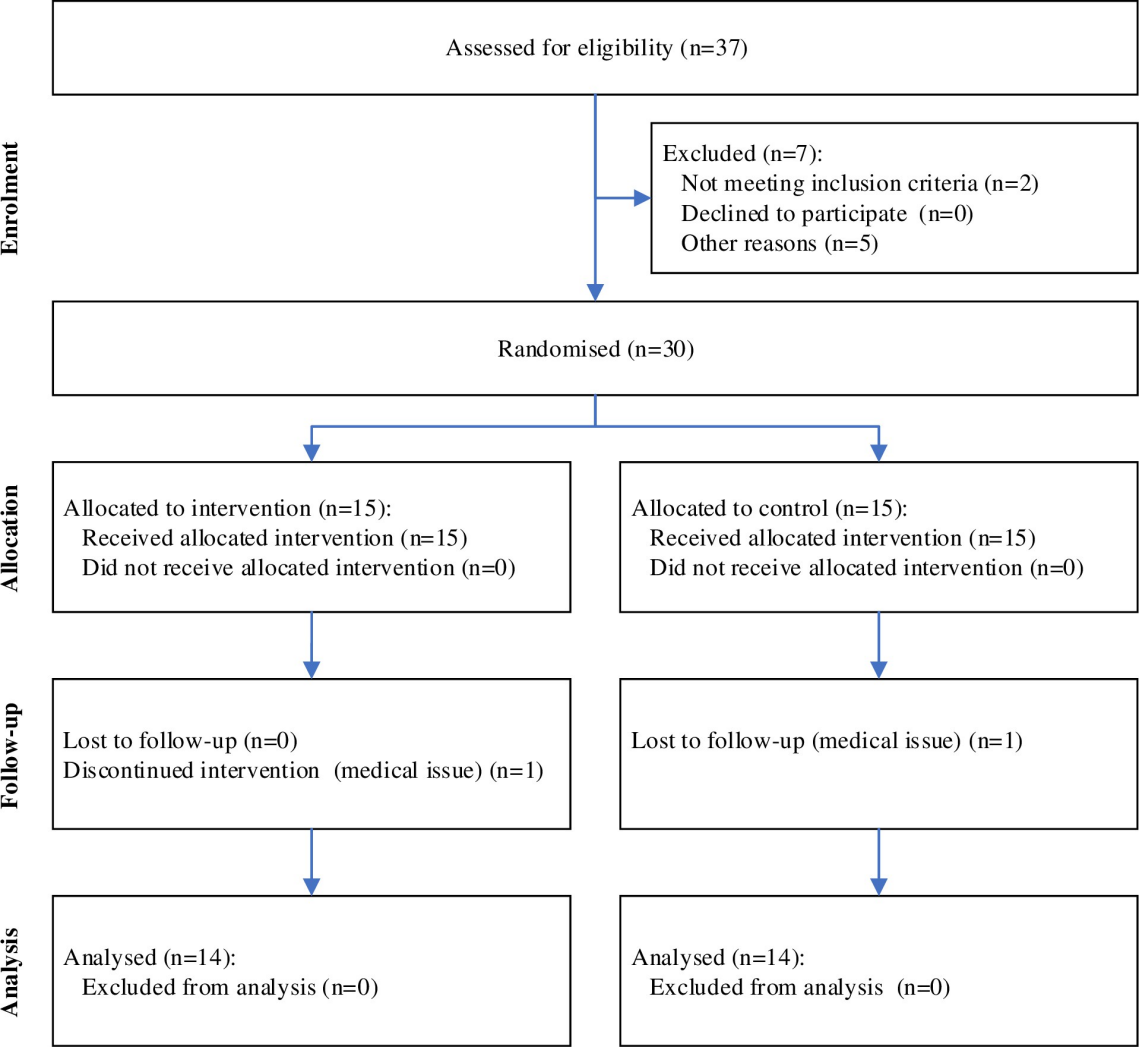
CONSORT flow diagram.

This study was a parallel-group, randomized, controlled trial with a 1:1 allocation ratio. We prospectively recruited participants between 18 to 40 years in the university setting via flyers, e-mail distribution lists, social media channels, and direct contact from 29 June to 8 August 2022 before the measurements started. The study flow diagram is presented in Fig 1. All measurements were finished by the 15 October 2022. After the inclusion criteria were checked, participants were randomly assigned to the control- or MS group via a computerized random sequence list generator [40] by the study lead, who was not involved in the recruitment process.

**Fig 2:**
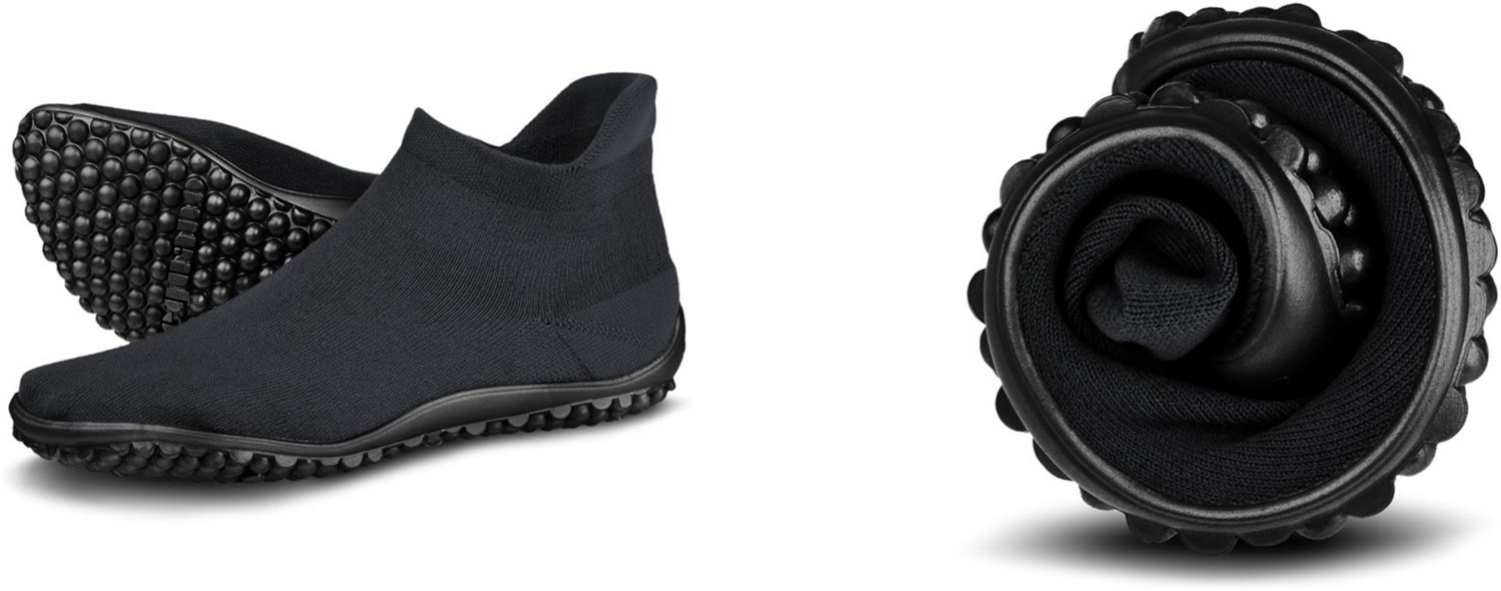
Minimalist shoe (Sneaker, Leguano GmbH, GER) worn by intervention group participants. Material: 53% polyamide, 38% lyocell, 7% polypropylene, 2% elastane. Sole: 100% LIFOLIT®. Reprinted from figures provided by the company under a CC BY license, with permission from Leguano GmbH, original copyright 2023.

Before measurement, participants received an e-mail in which they were instructed to avoid strenuous physical activity in the 24 hours preceding the test sessions. Participants were also required to watch a video to familiarize themselves with the measurement methods. They then visited the university’s laboratory for three measurement sessions (M1-M3), each approximately 60 minutes in duration. At the end of the baseline measurement session (M1), the study lead provided participants in the MS group with a pair of MS (Sneaker, Leguano GmbH, GER) (Minimalist index 96% (11)) (Fig 2) and informed them about the intervention (see 2.3). The first post-measurement (M2) was conducted after the four-week intervention at the same time of the day as M1. Following M2, participants in the MS group paused the intervention for four weeks before the third measurement (M3) (also same day time as M1 and M2).

### Participants and eligibility criteria

Participants were all classified as low to moderately active [41], based on a weekly average of at least 5,000–10,000 steps per day, recorded before the commencement of the study via a smartphone application (Accupedo, Corusen, USA). Participants were excluded from the study if they walked less than 5,000 steps/day over an average week, regularly wore MS, were professional athletes, regularly performed running, were pregnant, or were in a child-nursing period. We excluded persons with diagnosed (childhood) foot deformities, pain involving the trunk or upper or lower extremities, musculoskeletal injury involving the lower extremity or lower back in the last 12 months, a history of surgery in the lower limb or lower back, or self-reported impairment that would affect general motor function, balance, blood circulation, or pain sensation.

### Intervention

All participants were encouraged to maintain their pre-study level of walking and sports. Participants in the MS group were instructed to walk approximately a third of their usual daily steps (i.e., 2,500–3,000) with MS (Sneaker, Leguano GmbH, GER) (Fig 2) (Minimalist index 96% [11]) in the first week [32]. They were encouraged to perform the intervention daily, but at least five days per week, and to walk on various surfaces. After the first week, all participants were contacted via telephone to answer any potential questions and improve training compliance. If participants in the MS group reported no adverse events (e.g., pain), they were subsequently requested to increase their daily step count to a maximum of 5,000 [32]. Participants of the MS group tracked the number of steps taken in MS during the intervention period via a smartphone app (Accupedo, Corusen, USA). They documented them via web-based training software (Lanista, MP Sports, Coaching & Consulting GmbH, GER). The control group continued walking in their individually preferred shoe (no MS or lightweight shoe) according to their pre-study activity level.

### Outcomes

As described in the introduction, MS were reported to affect foot health [8, 9, 13, 14, 19, 24, 25, 32]. The outcome variables chosen in this study are commonly applied in studies investigating the foot and represent various dimensions of foot health: Foot Posture Index-6 (FPI-6) for foot posture [42], Arch Rigidity Index (ARI) for MLA rigidity [3], static single-leg stance balance and ARI both indirectly for IFM strength [24, 25, 43]. The local ROM parameters (i.e., first metatarsophalangeal joint (MTPJ1) and ankle) complete the comprehensive local foot assessments [19, 31]. Further, according to prior literature on remote effects along the PC after interventions on the plantar foot sole, we assessed the following remote or total PC parameters: ROM, modified back-saver sit-and-reach test [44–48]; strength, Bunkie Test [49], standing 90:20 Isometric Posterior Chain Test (IPCT) [50, 51], and isokinetic hamstring strength [52]. In each session (M1-M3), participants warmed up on a cycle ergometer (80 W) at a self-selected speed and then performed the tests in the following order: FPI-6, ARI, static single-leg stance balance, ROM MTPJ1, ankle, and PC, Bunkie Test, IPCT, isokinetic measurement. All tests were examined by experienced personnel (physiotherapists or sports scientists). The same experienced physiotherapist always assessed the FPI-6 and the Bunkie Test. The test sequence between limbs was randomized based on limb dominance (preferred leg for kicking a ball) via an online random sequence generator [40].

#### Foot parameters

*Foot posture index 6*. The FPI-6 [53] includes six parts, which evaluate the forefoot and rearfoot components in the three cardinal body planes. The scoring system uses a 5-point Likert-type scale where lower scores represent a more supinated foot and higher scores a more pronated position. For further analysis, we changed the original scoring system for each component (−2 to 2) to the adapted score (1 to 5) [54], where 3 indicates a neutral foot position. Participants were instructed to jump three times before measurement and then stand relaxed.

*Arch rigidity index*. The Arch Height Index was determined from caliper measurements of each foot made during a quiet one-leg stance. Measurements were performed with a custom-built measurement system, as reported by Mulligan and Cook [55] and Tourillon, Gojanovic [56]. For the seated Arch Height Index, participants sat on a chair with their hips and knees flexed to 90° with their test foot positioned on the measurement system such that the posterior aspect of the heel made contact with a fixed bar. The foot was then manually placed in a subtalar joint neutral position, defined as a symmetrical position of the talar head within the tarsus on palpation. The foot length was measured from the posterior heel to the longest toe. A marker was set at 50% of the foot length, from where the arch height was measured with a caliper (modified carpenter square). The Arch Height Index was calculated by dividing the height of the dorsum of the foot by the length of the foot [56]. All measurements were repeated as participants stood on the measurement system. The examiner checked and corrected potential hip abduction during standing. The ARI, which represents the structural rigidity of the MLA [55], was calculated as the ratio of the Arch Height Index determined during single-leg stance to that during sitting.

### Static single-leg stance balance

For the balance measurement, the center of pressure (CoP) was investigated during a static single-leg stance. Participants were instructed to stand on a pressure measurement system (Footscan, Gait and Motion Technology Ltd, UK) with the eyes open, looking straight ahead, and the knee of the contralateral limb flexed to approximately 60°. Pressure data were recorded at a sampling rate of 300 Hz for 25 s with a five-s delayed start to control for initial balance irritations. Then, after a 30-s pause, in which participants were allowed to take a few steps, the measurement was repeated on the contralateral limb [57, 58]. We evaluated the distance of the CoP in mm (CoP path) and the ellipse area of the CoP in mm^2^ (CoP EA).

### Range of motion

*Range of motion of the first metatarsophalangeal joint*. For the assessment of MTPJ1 ROM, participants performed a lunge test and were instructed to lift the heel of the tested limb as far as possible while maintaining contact with MTPJ1 to the support surface [59]. A clinical goniometer (Model 01135, Lafayette Instruments Co., Sagamore, IN, USA) was then used to measure the angle formed by the shaft of the first metatarsal and the support surface to the nearest degree [59, 60]. Participants were allowed to hold on to the wall and bend the knee of the lead and trailing limb during the test. The movement was repeated three times, and the maximum ROM was recorded.

*Range of motion ankle joint*. Ankle ROM was assessed with the knee-to-wall test according to the established protocol applied in prior studies [61]. The test is a commonly used clinical tool to measure ankle dorsiflexion ROM and is also often referred to as Weight-Bearing-Lunge-Test [61]. Participants were instructed to align their heel and hallux along a standard tape measure, with their toe 10 cm from the wall. They were asked to bend the knee in line with the second toe, with the goal of touching the wall with the knee (i.e., vertically fixed tape). Participants were allowed to hold on to the wall. We further standardized the test (i.e., lifted heel) by placing a tightened elastic band (TheraBand, Ludwig Artzt GmbH, GE) under the participants’ heel with one end fixed on the ground. If participants could touch the wall with their knee, their foot was progressively placed further from the wall in 1-cm increments. The lunge was repeated until participants could not touch the wall with their knee without lifting the heel off the ground. Ankle ROM was defined as the distance of the hallux from the wall, measured to the nearest cm.

*Flexibility total posterior chain*. The modified back-saver sit-and-reach test is an alternative to the classical sit-and-reach test, commonly applied to assess unilateral hamstring and lower back flexibility [62, 63]. It allows one leg to be tested at a time and considers leg length discrepancies and discomfort in the contralateral hip joint [64]. For the test, participants sat on a bench with a height of approximately 30 cm and placed the foot of the tested leg on a standardized sit-and-reach box. The untested leg was placed on the floor with the knee flexed approximately 90°. Three trials were performed per leg, where the best score was used for further analysis.

### Muscle strength of the posterior chain

*Standardized bunkie test*. For the standardized Bunkie Test for the PC, participants placed their forearms on a mat in a supine position, with their shoulders over their elbows and their heels on a box (height, 30 cm), with both legs straightened [65, 66]. To assess one leg, participants lifted the pelvis to a neutral position and then raised the contralateral leg approximately 10 cm off the box. For standardization, the horizontal position of the pelvis was marked with a rubber band, and the height of the lifted contralateral limb was defined by a box (height 10 cm). The duration that the participant could maintain the correct position was recorded using a stopwatch (in seconds). The test was ended if participants reported burning, cramping, pain, or strain, stopped the test due to fatigue, or reached the cutoff score (40 s) [66]. If they could not maintain the test position, the examiner verbally corrected them and allowed them to correct the position once. If there were any further deviations, the test was ended. After a 30-s pause, the test was repeated for the contralateral limb [66–68].

*Standing 90*:*20 isometric posterior chain test*. For the IPCT, participants stood with their legs and lower back against a wall with arms across and their hands placed on the opposite acromion. The tested leg was placed on a force plate (i.e., ankle loose and neutral) (FP4060-10-TM-2000, Bertec, Columbus, Ohio, USA) fixed to a height-adjustable desk. The height of the force plate was individually adjusted so that participants had 90° of hip flexion and 20° of knee flexion position, verified with a goniometer. Participants were instructed to place the hand on the contralateral shoulder and keep their standing leg extended and in contact with the wall, which was visually confirmed by the examiner [50, 51].

The examiner gave the instruction "to exert maximal force vertically into the force plate (i.e., the ground)" and continued verbal motivation (ca. 5 s). Each limb was tested alternately three times with a 30-s pause between. The force plate was connected to a 16-bit A/D converter, and the sampling frequency was set at 1,000 Hz with no filter. Force values (N) were captured with proEMG (prophysics AG, CH). Force data were smoothed with a 20-ms moving average.

For data analysis, we extracted the maximum vertical force of the three trials for each leg.

*Isokinetic measurement of the hamstrings’ strength*. The strength of the knee flexors during concentric movement (ROM 5°–90°) was evaluated via isokinetic testing. Participants were seated on a dynamometer (ISOMED 2000, D & R Ferstl GmbH, GER) with their hips in 90° flexion and fixed with straps over the middle of the thigh, waist, and shoulders. The axis of rotation of the dynamometer arm was aligned with the femoral epicondyle. Five repetitions were performed at 60°/s and 120°/s with a 1-min pause between each repetition. Before each trial, participants completed a test trial with five repetitions at sub-maximal effort to warm up and familiarize themselves with the movement and angular velocity. The test was repeated for the other leg [69–72]. The angle and torque values (Nm) were captured with proEMG at 1000 Hz (prophysics AG, CH) and processed in Matlab (R2020b, MathWorks, USA). We calculated the average of the maximum flexion peak torque (i.e., repetition 2–4) for both angular velocities.

### Blinding

The examiners were blinded concerning the study group allocation, and participants were not told about the other groups’ tasks during the study.

### Sample size

We estimated the sample size with G*Power version 3.1. (Heinrich Heine University, Düsseldorf, Germany). In the absence of MS studies involving recreationally active participants (non-runners), we based our sample size calculation on a study investigating the effects of another foot health intervention, namely IMF exercises. The calculation was based on one of the primary outcome variables, namely foot posture (i.e., FPI-6). Based on the results of a prior study for an IFM intervention duration of six weeks, we assumed a within-group standard deviation (*sd*) of 1.5 points and a relevant difference between the groups of 2 points [73], translating to an effect size of Cohen’s *d* = 1.3. With *d* = 1.3 converted to Cohen’s *f* = 0.7 [74] (for ANOVA) and assumed *α* = .05 and *β* = .95, the estimated sample was n = 30 in total, which resulted in n = 15 per group. Our calculated sample appears reasonable according to a prior study investigating a foot health intervention, similar to this study concerning duration and population [75].

### Statistical methods

Statistical analysis was performed using the statistical software R (version 3.5.1, R Core Team, AUT) [76]. For the analysis of the outcome variables, we first checked the required assumptions. We excluded data due to sampling or measurement problems (S2 Appendix). For continuous outcome variables, the mean of both legs was used. We fitted multiple hierarchically built mixed models to the data using the lme4 package [77]. Repeated measures (time effect) were nested within the variable participant, which was specified as a random factor (random intercept). We built the models adding subsequently the following predictors: (1) intercept-only; (2) measurement (time) as predictor variable; (3) measurement and intervention as predictor variables; (4) main effect of both variables as well as their interaction term as predictors. The models were compared via analysis of variance (ANOVA), and regression coefficients with corresponding 95% confidence intervals and standard errors (SE) are reported. A *p*-value of ≤ .05 was considered significant.

## Results

Descriptive statistics of outcome parameters and removed outliers are listed in S1 Appendix A Table 1 and S2 Appendix. All relevant data are within the manuscript.

**Table 1:** Participants’ characteristics at baseline.

Variable (mean ± *sd*)	Total *n* = 28	Minimalist shoe *n* = 14	Control *n* = 14
Age (years)	25.3 ± 5.3	26.1 ± 5.8	24.5 ± 4.8
Weight (kg)	70.2 ± 11.9	70.2 ± 10.1	70.2 ± 13.8
Height (cm)	175.0± 7.8	175.0 ± 7.7	175.0 ± 8.2
Steps/day (n)^#^	8,450 ± 2,516	7,723 ± 2,341	9,177 ± 2,555
(*n* =)			
Sex	15 f/13 m	7 m/7 f	6 m/8 f
Dominant leg	5 left/23 right	14 right	5 left/9 right

*Note*. f, female; m, male; sd, standard deviation

^#^ average steps/day over a regular week were tracked by participants via smartphone app (Accupedo, Corusen, USA) before study inclusion.

### Participants

Two female participants (one control group and one MS group) dropped out of the study after M1 due to acute medical incidents. Consequently, the final sample consisted of 28 participants. There were no differences between groups for any measured variable (Table 1) (all *p* > .05). Participants in the MS group reported that they walked on average (mean ± *sd*) 2,927 ± 1,891 steps/day in the first week and 3,955 ± 1,117 steps/day in weeks two to four. During weeks two to four, participants in the MS group walked the maximum of 5,000 steps/day on 30% of the intervention days and did at least a third of the 5,000 steps on 70% of the days. The MS group reported no adverse or unwanted side effects (i.e., pain) at direct telephone contact during the intervention.

### Foot parameters

**Fig 3:**
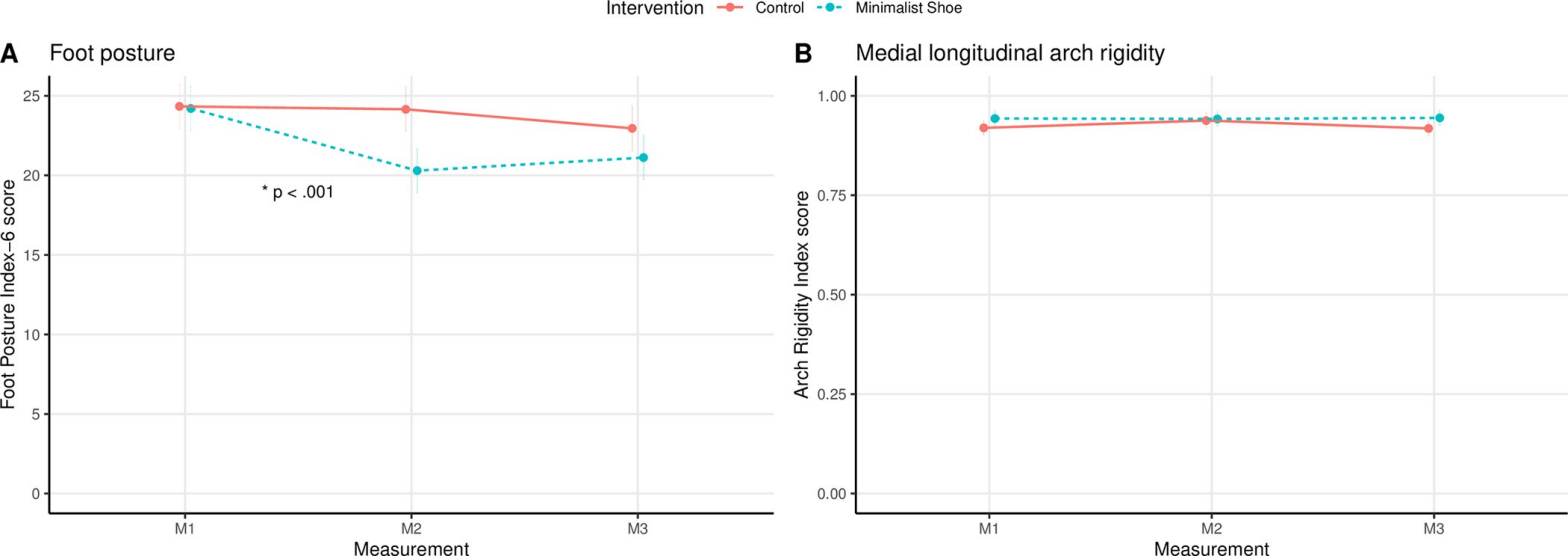
Effect plot (mean ± standard error) of the intervention and measurement time point for the foot parameters: (A) Foot Posture Index-6, and (B) Arch Rigidity Index. Note. * indicates a significant interaction (*p* ≤ .05).

The results of the linear regression models are presented in Table 2. The effect plots of the mean ± SE over time for both groups and the interaction between groups over time for the foot parameters (i.e., FPI-6, ARI) are shown in Fig 3.

**Table 2:** Results of the linear regression models of the outcome parameters for the minimalist shoe and control group representing changes from baseline (M1) to the second (M2) and third (M3) measurement time points.

	b	p-value	SE b	95% CI
FPI-6				
Control M2	-0.18	.68	0.44	-1.04, 0.68
Control M3 *	-1.38	.003	0.45	-2.26, 0.50
MS M2 *	-3.72	< .001	0.62	-4.94, 2.51
MS M3 *	-1.71	.009	0.62	-2.94, 0.48
ARI				
Control M2	0.018	.13	0.012	-0.005, 0.042
Control M3	-0.001	.91	0.012	-0.025, 0.022
MS M2	-0.019	.24	0.017	-0.052, 0.013
MS M3	0.003	.86	0.017	-0.030, 0.035
CoP path				
Control M2	-7.07	.72	19.66	-45.80, 31.65
Control M3	2.12	.92	20.20	-37.68, 41.93
MS M2	-37.73	.17	27.27	-91.45, 16.00
MS M3 *	-69.33	.02	27.56	-123.57, 15.08
CoP ellipse area				
Control M2	13.43	.009	4.90	3.95, 22.91
Control M3	5.15	.31	5.03	-4.57, 14.88
MS M2 *	-17.96	.01	7.02	-31.54, 4.37
MS M3 *	-15.97	.03	7.11	-29.72, 2.21
ROM MTPJ1				
Control M2 *	-7.01	.02	2.98	-12.88, 1.13
Control M3 *	-6.29	.05	3.06	-12.33, 0.26
MS M2	4.56	.28	4.21	-3.75, 12.87
MS M3	-0.15	.97	4.27	-8.57, 8.27
ROM Ankle				
Control M2	0.17	.67	0.39	-0.59, 0.93
Control M3	-0.31	.45	0.40	-1.09, 0.48
MS M2	-0.03	.96	0.55	-1.11, 1.05
MS M3	0.02	.97	0.56	-1.08, 1.11
ROM posterior chain				
Control M2	1.36	.07	0.72	-0.06, 2.78
Control M3	0.70	.35	0.74	-0.76, 2.16
MS M2	0.60	.57	1.02	-1.40, 2.61
MS M3	1.03	.32	1.03	-1.01, 3.06
Bunkie Test				
Control M2	0.79	.71	2.16	-3.46, 5.05
Control M3 *	6.56	.005	2.21	2.20, 10.93
MS M2 *	6.97	.03	3.11	0.84, 13.10
MS M3	2.89	.36	3.15	-3.31, 9.10
IPCT				
Control M2 *	-15.98	.03	7.16	-30.09, 1.86
Control M3	8.44	.25	7.31	-5.98, 22.85
MS M2 *	21.14	.04	9.93	1.55, 40.74
MS M3	12.30	.23	10.04	-7.51, 32.12
Isokinetic measurement of hamstrings’ strength at 60°/s				
Control M2	-3.25	.24	2.70	-8.58, 2.08
Control M3	-4.20	.13	2.70	-9.53, 1.13
MS M2	-2.56	.50	3.77	-9.99, 4.87
MS M3	-2.87	.45	3.77	-10.30, 4.57
Isokinetic measurement of hamstrings’ strength at 120°/s				
Control M2	-3.27	.21	2.58	-8.35, 1.82
Control M3	-1.41	.59	2.58	-6.49, 3.68
MS M2	-0.11	.98	3.60	-7.20, 6.98
MS M3	-4.48	.23	3.60	-11.57, 2.61

*Note*. ARI, Arch Rigidity Index; CI, confidence interval; FPI-6, Foot Posture Index 6; M2, measurement point 2; M3, measurement point 3; MS, minimalist shoe group; SE, standard error; ROM, range of motion; MTPJ1, first metatarsophalangeal joint; CoP, center of pressure; IPCT, standing 90:20 Isometric Posterior Chain Test; Note

* indicates a statistically significant result *(p* ≤ .05*)*.

The comparison of the linear models via ANOVA revealed a significant difference in FPI-6 score over time (*χ*^2^(2) = 30.21, *p* < .001) and between groups (*χ*^2^(1) = 3.63, *p* = .05). Furthermore, there was a significant interaction between groups over time for the FPI-6 score (*χ*^2^(2) = 29.63, *p* < .001). There was a significant improvement in the FPI-6 score of 16.9% from M1 (mean ± *sd*: 24.2 ± 2.0) to M2 (20.1 ± 2.1) (*p* < .001) and of 13.6% to M3 (20.9 ± 2.3) (*p* = .009) for the MS group (Table 2). For the control group, there was a significant improvement of 4.1% between M1 (24.3 ± 3.6) and M3 (23.3 ± 3.3) (*p* = .003), but not between M1 and M2 (24.5 ± 2.9) (Table 2).

The ANOVA showed that the time point of the measurement had no significant effect on the ARI score (*χ*^2^(2) = 1.28, *p* = .53). In contrast, there was a significant main effect of group (*χ*^2^(1) = 4.16, *p* = .04). The interaction between groups over time was not significant for ARI (*χ*^2^(2) = 2.13, *p* = .35). The linear model for the ARI showed no significant effects.

### Static single-leg stance balance

**Fig 4:**
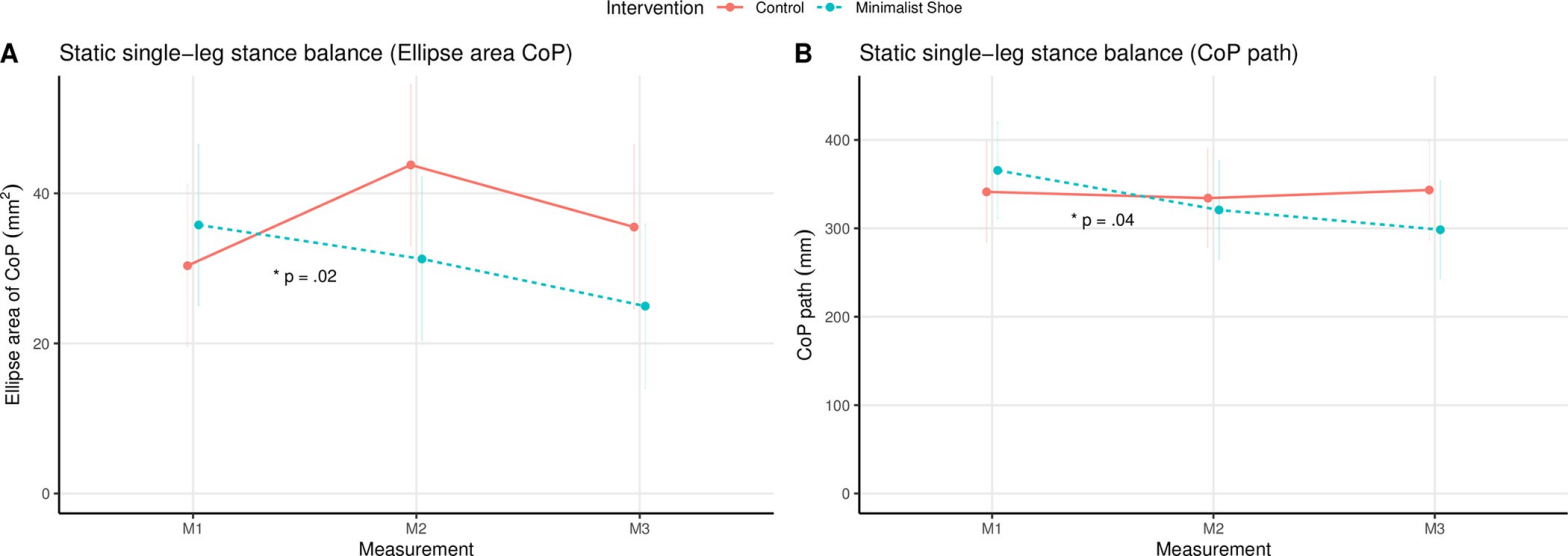
Effect plot (mean ± standard error) of the intervention and measurement time point for the static single-leg stance balance parameters: (A) ellipse area CoP, and (B) CoP path. Note. CoP, Center of pressure; * indicates a significant interaction (*p* ≤ .05).

The results for the total linear models are listed in Table 2. The effect plots of the mean ± SE over time for both groups and the interaction between the groups over time for the static single-leg stance balance parameters (i.e., CoP path, CoP EA) are shown in Fig 4.

The comparison of the linear models via ANOVA revealed a significant difference over time (*χ*^2^(2) = 6.28, *p* = .04) and an interaction between groups over time (M1 to M2) (χ^2^(2) = 6.36, *p* = .04) for CoP path, while the main effect for group was not significant (χ^2^(1) = 0.08, *p* = .78). There was a decrease of 14.4% in CoP path from M1 (361 ± 145 mm) to M2 (309 ± 124 mm) and of 6.1% to M3 (290 ± 91 mm) in the MS group, which became only significant at M3 (*p* = .02) (Table 2). For the controls, there was an increase of 3.3% from M1 (332 ± 87 mm) to M2 (343 ± 130 mm) and of 4.4% to M3 (358 ± 131 mm), yet not significant (Table 2).

Both the main effect for time and group were not significant for the CoP EA (measurement: *χ*^2^(2) = 4.00, *p* = .14; group: χ^2^(1) = 0.72, *p* = .40), whereas the interaction of the intervention between the groups over time was significant (*χ*^2^(2) = 7.94, *p* = .02). For the CoP EA, there was a significant decrease of 13.9% from M1 (36 ± 19 mm^2^) to M2 (31 ± 18 mm^2^) (*p* = .01) and of 33.3% to M3 (24 ± 8 mm^2^) (*p* = .03) in the MS group (Table 2).

### Range of motion

**Fig 5:**
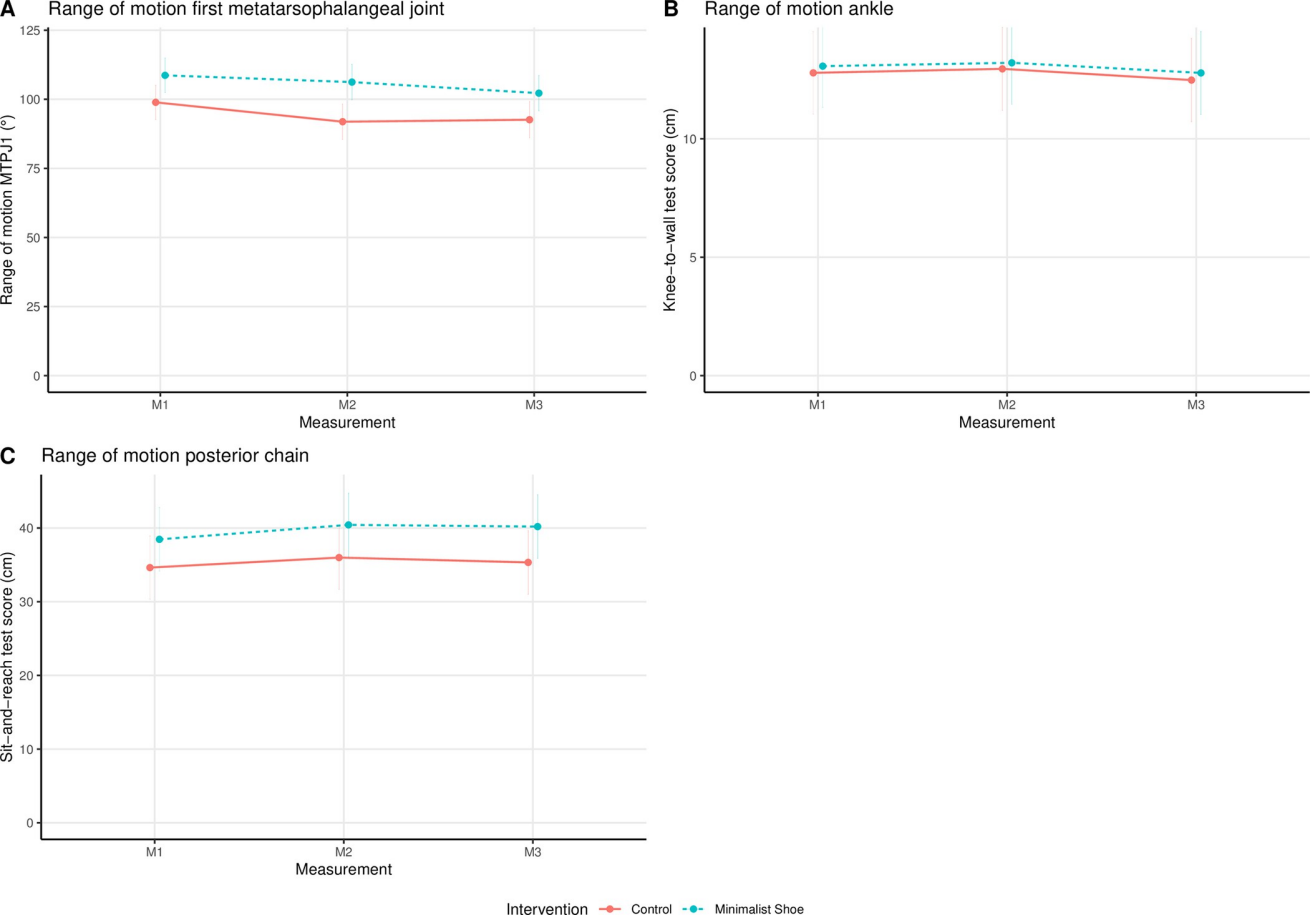
Effect plot (mean ± standard error) of the intervention and measurement time point for the range of motion parameters: (A) first metatarsophalangeal joint, (B) ankle, and (C) posterior chain. Note. MTPJ 1, first metatarsophalangeal joint.

The results for the total linear models are listed in Table 2. The effect plots of the mean ± SE over time for both groups and the interaction between the groups over time for the ROM parameters (i.e., MTPJ1, ankle, total PC) are shown in Fig 5.

There was a significant main effect of time (*χ*^2^(2) = 9.00, *p* = .01) and group (χ^2^(1) = 7.34, *p* = .007) on MTPJ1 ROM, whereas the interaction between groups over time was not significant (*χ*^2^(2) = 1.62, *p* = .44).

For the PC ROM, the time point of the measurement was significant (*χ*^2^(2) = 10.55, *p* = .005). In contrast, for the ankle ROM, it was not (*χ*^2^(2) = 2.77, *p* = .25). For both total PC and ankle ROM, the ANOVA showed no statistically significant main effect for group (ankle: χ^2^(1) = 0.05, *p* = .82; PC: χ^2^(1) = 1.98, *p* = .16) or interaction (ankle: *χ*^2^(2) = 0.01, *p* = 1.00; PC: *χ*^2^(2) = 1.03, *p* = .60).

### Muscle strength of the posterior chain

**Fig 6:**
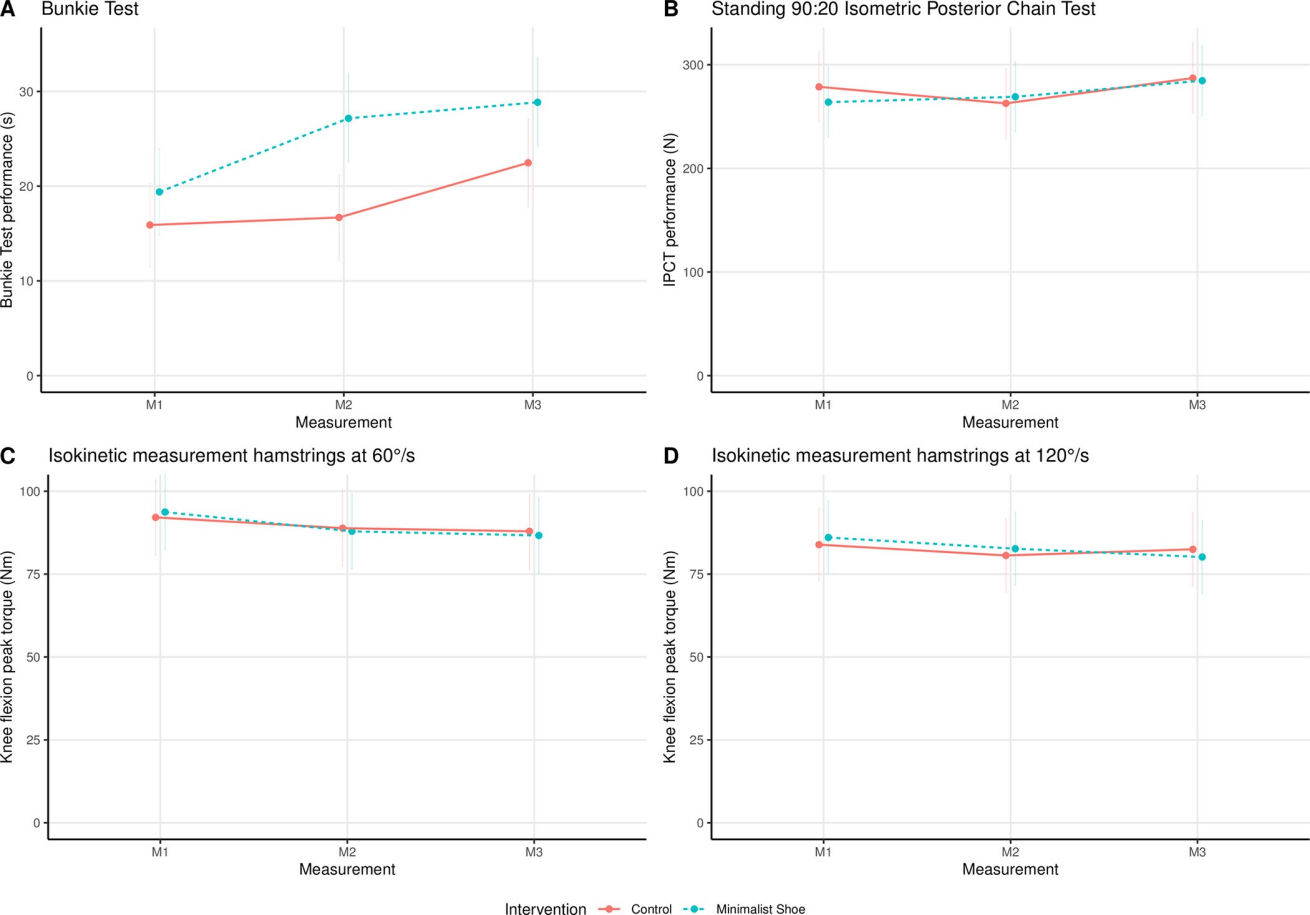
Effect plot (mean ± standard error) of the intervention and measurement time point for the remote strength parameters: (A) Bunkie Test, (B) IPCT, (C) Hamstrings 60°/s, and (D) Hamstrings 120°/s. Note. IPCT, standing 90:20 Isometric Posterior Chain Test; Hamstrings, isokinetic measurement of the hamstrings’ strength.

The results for the linear models are listed in Table 2, and the effect plots of the mean ± SE over time for both groups as well as the interaction between groups over time for the PC strength parameters (i.e., Bunkie Test, IPCT, hamstrings strength) are shown in Fig 6.

The main effect of the time point of the measurement (*χ*^2^(2) = 20.23, *p* < .0001) and the group (*χ*^2^(1) = 5.38, *p* = .02) had a significant effect on Bunkie Test performance. In contrast, the interaction of groups over time showed no significant result (*χ*^2^(2) = 5.00, *p* = .08).

For the IPCT, only the time point of the measurement was significant (*χ*^2^(2) = 13.72, *p* = .001), whereas the main effect of group (*χ*^2^(1) = 0.04, *p* = .85) and the interaction (*χ*^2^(2) = 4.60, *p* = .10) were not significant.

The time point of the measurement showed a significant main effect on isokinetic hamstring strength at 60°/s (*χ*^2^(2) = 9.43, *p* = .009). In contrast, at 120°/s it did not (*χ*^2^(2) = 4.88, *p* = .09). For both angular speeds (i.e., 60°/s and 120°/s), the main effect of group (60°/s: *χ*^2^(1) = 0.00, *p* = .99; 120°/s: *χ*^2^(1) = 0.01, *p* = .92) and interaction between groups over time (60°/s: *χ*^2^(2) = 0.73, *p* = .70; 120°/s: *χ*^2^(2) = 2.02, *p* = .37) showed no significant effects.

## Discussion

The present study investigated the effects of a four-week MS walking intervention on foot parameters (i.e., foot posture and MLA rigidity), static single-leg stance balance, local and composite ROM of the PC, and muscle strength of the PC. The main finding of our study is that walking in MS for four weeks may improve foot shape from a more pronated position toward a neutral foot position in healthy participants, resulting in an improved FPI-6 score at M2, which could also be maintained after a four-week intervention pause. Moreover, the MS group showed improved static single-leg stance balance at M2 and M3 (yet the CoP path at M2 was not significant). The novelty of our study is the assessment of the long-term effects of the intervention after a four-week wash-out period. Our study is one of the few that assessed the local and, additionally, the remote influence of MS walking intervention among healthy, recreationally active young adults.

Except for the foot posture, other foot parameters showed no significant results. There were also no significant differences in the ROM parameters between groups. The composite strength measurements of the PC (i.e., Bunkie Test and IPCT) and the isolated isokinetic hamstrings’ strength measurement showed no significant interaction between groups over time. Therefore, we can only partly confirm our hypotheses, as four weeks of walking in MS positively affected foot posture (i.e., FPI-6) and IFM function (indirectly measured via static single-leg stance balance). Still, we could not see any effects on MLA rigidity (i.e., ARI), foot and ankle ROM, or any remote effects concerning PC ROM and strength in healthy participants.

Following the existing literature, it should be highlighted that in addition to the positive effects, adverse side effects, such as muscle pain or injuries, could occur when MS walking is not introduced in a controlled fashion [4]. Furthermore, it must be noted that walking in MS is not equal to barefoot walking [9] but could be a suitable alternative for urban habitats or when anti-slip soles are recommended.

### Foot parameters

Previous research has shown that wearing MS (for eight weeks or longer) is associated with improved IFM strength compared to baseline or non-MS-shoed populations [3, 17–19]. Although prior studies agreed that IFM are essential for stabilizing and influencing the MLA [7, 19], we found no changes in MLA rigidity in this study. Nevertheless, the measure of foot posture (i.e., FPI-6) improved. Miller, Whitcome [3], Davis, Hollander [4], Curtis, Willems [8] all suggest that the MLA deforming mechanisms might be improved by wearing MS. This seems logical due to the altered strike pattern and shoe flexibility in MS compared to cushioned shoes. Nevertheless, prior studies also found no significant influence of MS on the static MLA parameters (i.e., Arch Height Index, navicular height, and navicular drop) [3, 12]. According to these and our studies’ findings, one could discuss if the IFM have the strength capacity to increase the height of a fully loaded MLA during static stance. Moreover, for an increase in muscle strength to affect the MLA shape, it must be assumed that the muscles are inherently weak and unable to maintain the MLA in the first instance.

Nevertheless, although the MLA does not seem to change when wearing MS, the literature suggests that wearing MS influences general foot posture and leads to fewer foot deformities. D’AoÛt, Pataky [12] report that populations wearing MS show shorter and wider feet and a greater foot area. Further, the hallux angle decreases with the habitual use of MS [13, 14]. One could argue that, as the mid- or forefoot strike pattern applies more pressure to the forefoot, especially to the MTPJ [7, 10], it seems logical that static foot posture changes could instead be seen on the frontal plane or the forefoot, than in the sagittal plane (i.e., MLA) alone [5, 6]. This hypothesis is supported by prior studies, which found that the IFM growth is mainly seen in the muscles, which are prominent in the forefoot, primarily the short toe flexors, abductor digiti minimi, and abductor hallucis [3, 7, 8, 17–19]. This could be a potential explanation for why, in our study, we saw changes in the overall foot posture (measures all three body planes) but not the MLA alone. Further, at first glance, it seems strange that there were also significant changes in the FPI-6 in the control group at M3. Looking at the descriptive data, it becomes evident that there was only a minor change from (mean ± *sd*) 24.5 ± 2.9 at M2 to 23.3 ± 3.3 at M3 (in contrast to the MS group (M1: 24.2 ± 2.0, M2: 20.1 ± 2.1)).

### Range of motion

There were no significant interactions between groups over time for the ROM parameters. Currently, there is conflicting evidence on the effect of MS on foot ROM. Willy and Davis [31] report more dorsiflexion in the ankle and more knee flexion at the foot strike, and also Davis, Hollander [4] state that due to the elevated heel of a cushioned shoe, the foot is placed in greater plantarflexion at the foot strike. In contrast, Miller, Whitcome [3] report that wearing MS decreased dorsiflexion at foot contact during running. Hollander, Heidt [78] also report in their review that MS running goes along with reduced ankle dorsiflexion. Nevertheless, it must be noted that there seems to be a difference between walking and running in MS, as walking requires the foot to go through a greater ROM because of the potential heel-to-toe walking rather than mid- or forefoot striking during running [32]. However, no ROM changes, neither locally in the foot nor remotely in other PC areas, were found in this study. In contrast, studies proposed that, for example, via stretching or foam rolling on the plantar surface, ROM in remote body areas along the PC could be acutely or chronically increased [38, 45, 46, 79].

### Static single-leg stance balance

Balance ability, commonly tested when investigating the effect of MS or foot-strengthening interventions, can be seen as a functional parameter for foot mobility and stability [25, 43]. We found a positive impact of wearing MS on static single-leg stance balance. Both parameters (i.e., CoP path, CoP EA) decreased from M1 to M2 (not significant for the CoP path) and even further at M3. This is in line with prior studies. For example, Petersen, Zech [9], report an increased local dynamic stability in younger and older adults when walking in MS (measured via a motion capture system). Cudejko, Gardiner [25] found that elderly participants with a history of falls were more stable (reduced CoP range in mm) during standing and walking in MS, and that wearing MS additionally is beneficial for fall prevention and increasing mobility. Walking or running barefoot or in MS generally seems positively correlated with balance skills and consequently lowers the risk of falls [4, 8, 9, 19, 25]. This improvement in stability is reported to be associated with an increase in IFM strength [8, 19]. Stimulation of plantar receptors of the foot during MS could also be an essential aspect of the improvement in single-leg stance assessment observed in our study [30].

### Muscle strength of the posterior chain

Although studies frequently reported the positive effects of MS on IFM strength [3, 18, 32], little is known about the chronic effects of MS walking interventions on motor performance (i.e., sports) in general and, more specifically, about muscle strength of the PC [78]. In general, to date, the effects of long-term stimuli (especially muscle activation) on the plantar foot sole and its impact on the remote strength of the PC are rare. The influence on remote strength along the PC after exercise could be of practical relevance for the field of sports and rehabilitation if, for example, the targeted body area (e.g., hamstring muscles) cannot be trained locally for a specific amount of time due to injury (e.g., muscle strain) and further performance decreases should be prevented.

We assessed PC strength and performance with three different tests, addressing either isolated structures (i.e., hamstrings) or more composite measures of PC strength. In our study, the MS intervention did not affect isolated hamstring strength. Although this study gives first hints for the potential influence of wearing MS on composite strength measurements of the PC (i.e., Bunkie Test, IPCT) (see Table 1 in S1 Appendix A), the results must be interpreted with caution. Nevertheless, our results lead to the assumption that it cannot be an increase in hamstring strength (alone). Therefore, our results highlight the need for functional performance tests considering the total PC as also mentioned in prior studies [80].

Wearing MS not only seems to stimulate the local stabilizing muscles (i.e., IFM) but also the extrinsic foot muscles (i.e., posterior and medial calf muscles), which leads to higher force generation [7, 33, 81]. This could be explained by the changes in foot strike pattern [4, 7, 33, 81]. Further running in MS was reported to affect remote PC muscle activity during running (i.e., hamstrings and gluteal muscles) [33, 35], which could not be observed regarding chronic hamstring muscles strength increases or total PC performance improvements in this study.

An altered loading might explain these remote effects during running observed by prior studies [33, 35], as running in MS changes walking patterns and biomechanics, which includes an altered foot strike, a decrease in step length, and an increase in cadence [39]. Participants wearing MS during walking and running adapt to various surfaces by adjusting their overall leg stiffness, which increases vertical ground reaction force [4, 39, 82]. The altered foot mechanics could also affect more proximal regions of the leg and might lead to altered muscle activation in these body areas [4, 31, 78, 83]. However, transmission effects due to anatomical linkage via the PC seem to be unrealistic according to our findings.

### Limitations

One of the main limitations of our study is that, although we hypothesize that some of our parameters might be influenced by IFM strength, we did not measure IFM strength directly. According to Johnson, Myrer [18], isolated IFM strength measurement remains challenging as commercial dynamometers are not practicable for testing IFM strength. This is why prior studies usually refer to IFM size changes. Measurement via magnetic resonance imaging is used for that, but it is expensive and often not accessible. Further, the IFM differentiation is complex [18]. This is why ultrasound imaging is often preferred for measuring IFM size. However, this measurement technique is often not accessible, requires particular expertise, and refers to muscle size alone, which can also only be seen as an indirect measure of IFM strength [18]. This is why we refer to more functional, indirect measures of IFM strength (MLA rigidity and balance) instead in this study, as these parameters are influenced by IFM strength.

For the static single-leg stance balance parameters, it is noticeable that the *sd* of the descriptive data (Table 1 in S1 Appendix A) is markedly different from the MS condition in some cases. This could suggest that the individuals within the group may have had very different responses to the MS intervention.

Further, we only asked participants to track and report the steps when they wore the MS. This compromise kept participants’ efforts as low as possible to increase compliance with reporting. Nevertheless, this does not allow us to report the daily amount of steps taken by the control group or the MS group when not wearing the MS, including the wash-out period. We acknowledge this as a significant limitation of the study. However, both groups reported similar steps/day at baseline (i.e., tracked before the study started) (Table 1) and were asked not to make significant changes in sports or step level during the study. The self-reported exercise adherence (see 3.1.) matches the requirements, considering that participants were asked to perform the intervention at least five days per week.

In addition, one could argue that the stimuli via MS in our study were too low, including the intervention period and the steps/day. According to Tudor-Locke, Craig [41], adults taking at least 5,000 steps/day (inclusion criteria) can be classified as non-sedentary. As we wanted to investigate the effects of MS in recreationally active participants and at the same time avoid that participants increased their usual step amount during the study, we instructed them to walk ‘up to 5,000 steps in MS’ per day (on at least five days per week) from week two to four, which is in accordance to a prior study by Ridge, Olsen [32]. The intervention period in previous studies ranged from three weeks, over eight weeks [32], ten weeks [18], and 12 weeks [3] to several months [7, 8]. Ridge, Olsen [32] report that there were already changes in outcome parameters by four weeks and assume that shorter interventions might also be successful. This was also seen in four-week foot strengthening interventions [52, 55, 75]. As we wanted to keep compliance high, participants’ burden low, and add a wash-out period, we decided on an intervention period of four weeks. In addition, we increased to 5,000 steps/day one week earlier than in the study by Ridge, Olsen [32]. Perhaps if our research had continued for a more extended period, the effect in the MS group could have been greater. Future studies should address this.

## Conclusion

This study showed a positive influence of a four-week walking intervention on foot posture and static single-leg stance balance in healthy young adults, which supports prior findings of other populations. These changes remained (i.e., foot posture) or even further improved (i.e., balance) after a four-week wash-out period. Besides that, four weeks of walking in MS had no significant effects on MLA rigidity, local and PC ROM, and remote strength in the PC. Walking with MS may be an advantageous method worth implementing into the daily routine of recreationally active young adults. In contrast to other interventions on the plantar foot sole (e.g., foam rolling), no positive chronic remote effects along the PC should be expected. Therefore, local training of PC muscles should be favored. Moreover, in the clinical setting, MS might be implemented as an add-on for the rehabilitation and prevention of orthopedic foot diseases or even suitable for other patient groups (e.g., balance disorders).

Nevertheless, as these conclusions are based on a healthy, young sample of a German population, more studies with various populations (e.g., age and patient groups) are needed to enable a general statement on the effectiveness of four weeks of MS walking. Further, more research is needed to assess the chronic remote strength and ROM effects of active interventions on the plantar surface on the PC. Future studies should include functional assessments that consider the total PC.

## Supporting information

S1 Checklist (PDF)

S1 Appendix A_effect of 4weeks MS walking. (DOCX)

S2 Appendix B_effect of 4weeks MS walking. (DOCX)

S1 File (PDF)
