# Supplementary material for: A four-week minimalist shoe walking intervention influences foot posture and balance in young adults–a randomized controlled trial
Source: PLoS One. 2024 Jun 20;19(6):e0304640. doi: 10.1371/journal.pone.0304640 (PMC11189255; doi:10.1371/journal.pone.0304640)
Supplement: S1 Appendix — (DOCX) [file pone.0304640.s002.docx]

**APPENDIX A**

**Table 1. Descriptive data of the outcome parameters for both groups (minimalist shoe and control) at all three measurement points (M1-M3).**

| Variable | Measurement | | Minimalist shoe  (mean ± sd) | Control  (mean ± sd) |
| --- | --- | --- | --- | --- |
| FPI-6 score | M1 | | 24.2 ± 2.0 | 24.3 ± 3.6 |
|  | M2 | | 20.1 ± 2.1 | 24.5 ± 2.9 |
|  | M3 | | 20.9 ± 2.3 | 23.3 ± 3.3 |
| ARI score | M1 | | 0.94 ± 0.04 | 0.92 ± 0.03 |
|  | M2 | | 0.94 ± 0.05 | 0.94 ± 0.05 |
|  | M3 | | 0.94 ± 0.04 | 0.92 ± 0.04 |
| CoP path (mm) | | M1 | 361 ± 145 | 332 ± 87 |
|  | | M2 | 309 ± 124 | 343 ± 130 |
|  | | M3 | 290 ± 91 | 358 ± 131 |
| CoP EA (mm^2^) | | M1 | 36 ± 19 | 30 ± 19 |
|  | | M2 | 31 ± 18 | 44 ± 31 |
|  | | M3 | 24 ± 8 | 37 ± 23 |
| ROM MTPJ1 (°) | | M1 | 109 ± 8 | 99 ± 16 |
|  | | M2 | 107 ± 8 | 92 ± 16 |
|  | | M3 | 103 ± 11 | 91 ± 15 |
| ROM ankle (Ktw score) | | M1 | 13 ± 4 | 13 ± 3 |
|  | | M2 | 14 ± 4 | 13 ± 3 |
|  | | M3 | 13 ± 4 | 13 ± 3 |
| ROM PC (SaR score) | | M1 | 38 ± 8 | 35 ± 11 |
|  | | M2 | 40 ± 7 | 35 ± 9 |
|  | | M3 | 40 ± 6 | 34 ± 10 |
| Bunkie Test (s) | | M1 | 19 ± 11 | 16 ± 7 |
|  | | M2 | 27 ± 10 | 17 ± 8 |
|  | | M3 | 29 ± 9 | 22 ± 11 |
| IPCT (N) | | M1 | 263.93 ± 57.10 | 278.70 ± 82.00 |
|  | | M2 | 273.49 ± 50.12 | 247.02 ± 56.26 |
|  | | M3 | 289.10 ± 44.42 | 292.00 ± 97.61 |
| Hamstrings 60°/s (Nm) | | M1 | 93.71 ± 23.27 | 92.10 ± 25.08 |
|  | | M2 | 90.64 ± 16.35 | 88.40 ± 26.02 |
|  | | M3 | 89.39 ± 18.79 | 87.62 ± 23.57 |
| Hamstrings 120°/s (Nm) | | M1 | 86.04 ± 21.66 | 83.88 ± 23.12 |
|  | | M2 | 85.33 ± 17.92 | 80.16 ± 23.11 |
|  | | M3 | 82.82 ± 17.89 | 82.39 ± 24.74 |
| *Note*. sd, standard deviation; FPI-6, Foot Posture Index 6; ARI, Arch Rigidity Index; M1; measurement point 1 (baseline); M2, measurement point 2; M3, measurement point 3; CoP, center of pressure; CoP EA, ellipse area of the CoP; ROM, range of motion; MTPJ1, first metatarsophalangeal joint; KtW, Knee-to-wall test; PC, posterior chain; SaR, modified back-saver sit-and-reach test ; IPCT, standing 90:20 Isometric Posterior Chain Test; Hamstrings, isokinetic measurement of the hamstrings’ strength | | | | |
