# Supplementary material for: A four-week minimalist shoe walking intervention influences foot posture and balance in young adults–a randomized controlled trial
Source: PLoS One. 2024 Jun 20;19(6):e0304640. doi: 10.1371/journal.pone.0304640 (PMC11189255; doi:10.1371/journal.pone.0304640)
Supplement: S2 Appendix — (DOCX) [file pone.0304640.s003.docx]

**APPENDIX B**

The excluded data for the three measurements (M1-M3) and the respective parameters are listed below.

**Foot parameters**

For the Arch Rigidity Index (ARI), we excluded the following data:

- Participant 23 (control group), M1, first tested leg (ARI: 1.03)

**Static single-leg stance balance**

For the center of pressure (CoP) path and the ellipse area (EA) of the CoP, we excluded the following data:

- Participant 18 (control group), M1, first tested leg (CoP path: 518)
- Participant 20 (control group), M1, first tested leg (CoP path: 592)
- Participant 25 (control group), M1, first tested leg (CoP path: 488)
- Participant 37 (MS group), M3, second tested leg (CoP EA: 67)

**Muscle strength of the posterior chain**

For the standing 90:20 Isometric Posterior Chain Test we excluded the following data:

- Participant 25 (control group), M2, first tested leg (450 N)
- Participant 29 (control group), M2, first (429 N) and second tested leg (469 N)
