# Supplementary material for: A four-week minimalist shoe walking intervention influences foot posture and balance in young adults–a randomized controlled trial
Source: PLoS One. 2024 Jun 20;19(6):e0304640. doi: 10.1371/journal.pone.0304640 (PMC11189255; doi:10.1371/journal.pone.0304640)
Supplement: S1 File — (PDF) [file pone.0304640.s004.pdf]

Transmission effects along the dorsal chain: direct and remote long-term effects of strength training of the intrinsic foot muscles, plantar foot stimulation, hamstrings strength training and stretching.

Content

1. Summary 4
2. Responsibilities 6
   a. Principle Investigator: 6
   b. Scientists involved: 6
   c. Institutions involved 6
   d. Financing 6
3. Background 7
4. Aims of the Study 8
   e. Primary outcome and/or Hypothesis 8
5. Outcomes 8
   a. Primary Outcome 8
   b. Secondary Outcomes 9
6. Study design 9
   a. Monocentric/multicentric 9
   b. Study arms: 9
   c. Randomisation 9
   d. Blinding 10
   e. Graphic representation 10
7. Study population 10
   a. Inclusion and exclusion criteria 10
   b. Number of study participants 11
   c. Means of recruitment 11
8. Order of study 11
   a. Methods of Study information and obtaining consent ..... **Fehler! Textmarke nicht definiert.**
   b. Intervention / Control 13
   C: Minimalist shoe walking 16
   c. Acquisition of Outcomes 17
   d. Temporal Sequence and duration of the study for the individual participant 22
   e. Total duration of the study 23
9. Risk-benefit analysis 23
   a. Benefit for the individual participant 23
   c. Burden and Risks for the individual participant 23

d. Abort criterion 23
e. Statement to medical tenability 24
10. Biometry 24
a. Confirmatory study 25
b. Explorative study 25
11. Data management and data protection 25
a. Acquisition, storing (Art Ort Dauer), data transfer, ensurement of data security 25
b. Anonymised / pseudonymised 26
c. Revocation, data deletion 26
12. Handling of biomaterial 26
13. Proband insurance 26
14. Signatures of principle investigator 27
15. References 28

1. Summary

**Title**	Transmission effects in the dorsal chain: direct and remote effects of strength training of the intrinsic foot muscles, plantar foot stimulation, hamstrings strength training and stretching.
**Institution**	[1]Technische Universität München<br>Fakultät für Sport- und Gesundheitswissenschaften<br>Professur für Konservative und Rehabilitative Orthopädie<br>[2] Institute of Human Movement Science, Sport and Health, Graz University, Austria
**Head of the study**	[redacted]
**Contributing authors**	[redacted]
**Hypotheses**	H0a: Strength training of the intrinsic foot muscles of the foot and toe flexors does not influence biomechanical parameters and performance locally and in non-directly adjacent areas of the dorsal chain.<br><br>H0b: Stretching of the hamstrings does not influence biomechanical parameters and performance locally and in non-directly adjacent areas of the dorsal chain.<br><br>H0c: Strength training of the hamstrings does not influence biomechanical parameters and performance locally and in non-directly adjacent areas of the dorsal chain.<br><br>H0d: Stimulation of the plantar foot (= minimalist shoe walking) does not influence biomechanical parameters and performance locally and in non-directly adjacent areas of the dorsal chain.<br><br>H0e: Stimulation of the plantar foot (= minimalist shoe walking), strength training of the foot muscles, strength training and stretching of the hamstrings have similar effects on the medial longitudinal arch mobility, foot strength and flexibility, performance and flexibility in the total dorsal chain and hamstrings strength.<br><br>H0f: Hamstrings stretching and hamstrings strength training have similar remote and local effects on medial longitudinal arch mobility, foot strength and flexibility, performance and flexibility in the total dorsal chain and hamstrings strength.
**Study subjects and exclusion criteria**	n=15 per group (total n=75)<br><br>Inclusion:<br>• healthy participants (m/w/d)<br>• aged 18-40 years<br>• Recreationally active<br>○ Which is defined as “participating in at least 20-60 minutes of moderate to vigorous intensity physical activity 3-5days/week”. Moderate intensity is typically defined as 3-6 METS (e.g. brisk walking) whereas vigorous is activities

	over 6 METS (jogging or running) (Beierle, Burton, Smith, Smith, & Ives, 2019)<br>○ and walking at least around 5000-10000 steps a day (=low to moderately active) (measured in the week before)<br><br>Exclusion:<br>• no consent to participate<br>• regular barefoot runners/walkers<br>• experienced with intrinsic foot muscles exercises<br>• professional athletes<br>• pregnancy<br>• diseases: diabetes mellitus/CRPS; childhood foot deformities; diagnosed flatfoot; pain in elbow, shoulders or foot; musculoskeletal injury or in the lower extremity in the last 12 months (incl. foot); history of surgery in the lower limb and foot; self-reported disability due to neurological/musculoskeletal impairment in the lower extremity, that makes participants unable to plantar-flex the toes e.g. or would affect motor function in general; osteoarthritis affecting the foot; plantar fasciitis; shin splits; any history of ankle or foot sprain, fracture in the leg or foot; neurological or vestibular impairment that affected balance; lumbosacral radiculopathy; soft tissue disorder such as Marfan or Ehlers-Danlos syndrome, vascular disorders that affect blood circulation in the lower extremity, skin disease in the foot area.
**Methods**	For the statistical analysis, the software R (R version 3.5.1, R Core Team, Austria) will be used.
**Relevance of the topic**	Transmission effects have been shown along the dorsal chain in prior studies. Various treatment techniques or exercises were shown to increase or reduce strength/performance or range of motion locally or in non-directly adjacent areas along the dorsal chain. This is relevant for several orthopedic disorders (e. g. plantar fasciitis), where non-directly adjacent structures, like the hamstrings were shown to be affected or correlated with. Frequently performed or recommended interventions in the field of sports and rehabilitation are foot strength exercises, hamstrings stretching and strengthening as well as minimalist shoe walking. They were shown to increase strength or range of motion right after the intervention locally. This study aims at investigating the long-term effects of these already investigated interventions on various outcome parameters (flexibility, strength/performance and foot posture), also in non-directly adjacent body areas of the dorsal chain.

2. Responsibilities

a. Principle Investigator:

Name [redacted]
Function and Institute

Street
Zip Code and City

b. Scientists involved:

Name [redacted]
Institute

Street
Zip Code and City

c. Institutions involved

None

d. Financing

The project is conducted as part of a PhD project of one of the contributing researchers. Therefore, we have no additional costs to declare.

3. Background

The current literature, investigating the local and remote effects of interventions on parts of the dorsal chain, reveals the structures of the foot as key component in influencing tensional states or functional performance of the foot itself or along the chain (Gabriel et al., 2021; Grieve et al., 2015; Sulowska, Mika, Oleksy, & Stolarczyk, 2019). Although the feet occupy only 5% of the areas of the human body, they control postures through afferent information obtained through the sense of the soles, provide stability for maintenance of balance, and absorb impacts (Kim & Kim, 2016). Stretching, active strength training and stimulation/minimalist shoe walking are commonly applied interventions in the field of orthopaedics and physiotherapy in terms of the investigation of transmission effects in the dorsal chain and in the treatment of orthopaedic disorders, like plantar fasciitis.

For example, studies showed, that self-massage and stretching of the plantar foot structures could increase flexibility along the dorsal chain, also in non-adjacent areas, like the hamstrings (Gabriel et al., 2021; Grieve et al., 2015). Similar to that, Wilke, Vogt, Niederer, and Banzer (2017) showed that stretching on the hamstrings increased range of motion in non-directly adjacent areas of the dorsal chain (neck). In addition, stretching of the plantar fascia is commonly recommended as a treatment strategy for patients with plantar fasciitis. Yet, poor extensibility of the gastrocnemius and hamstrings muscles was also shown to be associated with plantar fasciitis. The gastrocnemius and hamstrings muscles are connected with the plantar aponeurosis via the superficial backline. Therefore, studies recommend that not only stretching of the plantar fascia, but also of the muscles of the dorsal chain should be included in the treatment strategy (Ajimsha, Binsu, & Chithra, 2014). Self-massage and stretching on the plantar foot structures were also shown to might acutely decrease performance in the dorsal chain (Gabriel et al., 2021). Yet, it is unclear whether such a transmission effect could also be seen, if the stretching is performed on the hamstrings –as recommended for plantar fasciitis- on the plantar fascia and if there are also effects in long-term. Therefore, **one aim of this study is to investigate the long-termin remote effects of hamstrings stretching on local and remote foot strength and flexibility.**

In contrast, strength exercises, performed only on the foot, seem to increase muscle strength and power not only in the foot (Sarah T Ridge et al., 2018), but also in proximal segments (the hamstrings) of the dorsal chain in long-distance runners (Sulowska et al., 2019). This was, to our best knowledge, only tested in runners so far. These foot muscle exercises positively influence strength parameters of the foot. Therefore, this could be of high interest in athletes, but also in person with diseases (Tourillon, Gojanovic, & Fourchet, 2019). Strength training and stretching, in general, have been shown to have similar effects on range of motion (Afonso et al., 2021).

Although toe flexors strength is generated by a simultaneous action of both intrinsic (IFM) and extrinsic foot muscles, the IFM seem most likely to be the main contributors to metatarsophalangeal joints torque. Therefore, strengthening interventions should not be limited to extrinsic foot and ankle muscles (e.g., flexor digitorum longus, triceps surae, flexor hallucis longus), but should also target intrinsic foot muscles (Tourillon et al., 2019). Further, Sulowska et al. (2019) increased the complexity of the exercises by performing the exercise in a standing position, from which one could assume that the strength increase in the hamstrings might not be only the remote effect of strength increase in the foot muscles. Further, it is not clear, whether these increases are correlated with increase in foot muscle strength and changes in foot posture (Sarah T Ridge et al., 2018; Sulowska et al., 2019). **Therefore, the second aim will be to investigate the effect of foot muscles strength exercises (intrinsic and extrinsic) on medial longitudinal arch height, foot strength parameters in healthy, recreationally active participants. Further, in order to address the remote effect, participants should perform the exercises in a sitting position, and strength and flexibility parameters in the hamstrings region are assessed.**

In addition, the remote effect of strength training along the dorsal chain, should also be investigated in the opposite way, meaning it should be assessed if strength training of the hamstrings muscles also influences foot strength and flexibility parameters.

Similar positive effects on foot biomechanical and strength parameters were found for barefoot or minimalist shoe walking in runners (Sarah T Ridge et al., 2018). Yet, there is currently no evidence from studies for the effects of minimalist shoe locomotion on motor performance, also in non-direct adjacent areas, especially in non-runners (Hollander, Heidt, Van der Zwaard, Braumann, & Zech, 2017; Johnson, Myrer, Mitchell, Hunter, & Ridge, 2016; Sarah T Ridge et al., 2018). **Therefore, the third aim of the study is to investigate the local and transmission effects of barefoot walking on various parameters of structures of the dorsal chain (see above) in recreationally active participants.**

In order to contribute to the current body of knowledge, we additionally would like to investigate the follow-up effect, after a wash-out phase of 4 weeks post-intervention, and again, assess the potential beneficial or detrimental effects in a few participants after the wash-out phase. Our study should be one of the first, to compare the effect of the interventions, currently shown to be able to affect performance, muscle strength and flexibility in the dorsal chain.

4. Aims of the Study

e. Primary outcome and/or Hypothesis

H0a: Strength training of the intrinsic foot muscles of the foot and toe flexors does not influence biomechanical parameters and performance locally and in non-directly adjacent areas of the dorsal chain.

H0b: Stretching of the hamstrings does not influence biomechanical parameters and performance locally and in non-directly adjacent areas of the dorsal chain.

H0c: Strength training of the hamstrings does not influence biomechanical parameters and performance locally and in non-directly adjacent areas of the dorsal chain.

H0d: Stimulation of the plantar foot (= minimalist shoe walking) does not influence biomechanical parameters and performance locally and in non-directly adjacent areas of the dorsal chain.

H0e: Stimulation of the plantar foot (= minimalist shoe walking), strength training of the foot muscles, strength training and stretching of the hamstrings have similar effects on the medial longitudinal arch mobility, foot strength and flexibility, performance and flexibility in the total dorsal chain and hamstrings strength.

H0f: Hamstrings stretching and hamstrings strength training have similar remote and local effects on medial longitudinal arch mobility, foot strength and flexibility, performance and flexibility in the total dorsal chain and hamstrings strength.

5. Outcomes

a. Primary Outcome

- Toe and foot strength:
    - Pressure platform (RS Scan)

- Performance dorsal chain:
    - Bunkie Test
    - Standing isometric posterior chain test

- Strength hamstrings
    - Isokinetic measurement

b. Secondary Outcomes

- Foot posture: foot posture index (FPI-6); transversal arch/medial longitudinal arch and total foot length (RS Scan)
- Range of motion:
    - Ankle and toe range of motion
    - Modified backsaver sit-and-reach test (dorsal chain)

- Indirect foot strength:
    - Modified star excursion balance test, centre of pressure
    - Arch rigidiy index

6. Study design

a. Monocentric/multicentric

Monocentric.

b. Study arms:

- Intervention A: Strength training of plantar flexor and intrinsic foot muscles
- Intervention B: Hamstrings stretching
- Intervention C: Hamstrings strength training
- Intervention D: Stimulation of the plantar foot (= Minimalist shoe walking)
- Control: No intervention (E)

c. Randomisation

This study is a randomized controlled trial. For practical reasons, we will start the measurements with groups A, B and E only. Therefore, group randomisation will be performed between these three groups in the beginning, only. After the measurements of for intervention A and B and the control group are finished, we will continue participant recruitment for interventions C and D. For the second measurement block, randomisation will be performed between group C and D. Therefore, we will perform two computer generated sequence random allocations for the measurement block 1 (A,B,E) and 2 (C,D) (Figure 2), separately.

Further, in order to control for learning effects. The first leg tested in all of the measurements for all tests, will be subsequently changed between the dominant and the non-dominant leg within each group. Meaning the first participant of group A will get the dominant leg tested first and the non-dominant second and for the second participant of group A, the non-dominant leg will be tested first.

d. Blinding

The participants are blinded concerning the hypotheses of the study.

e. Graphic representation

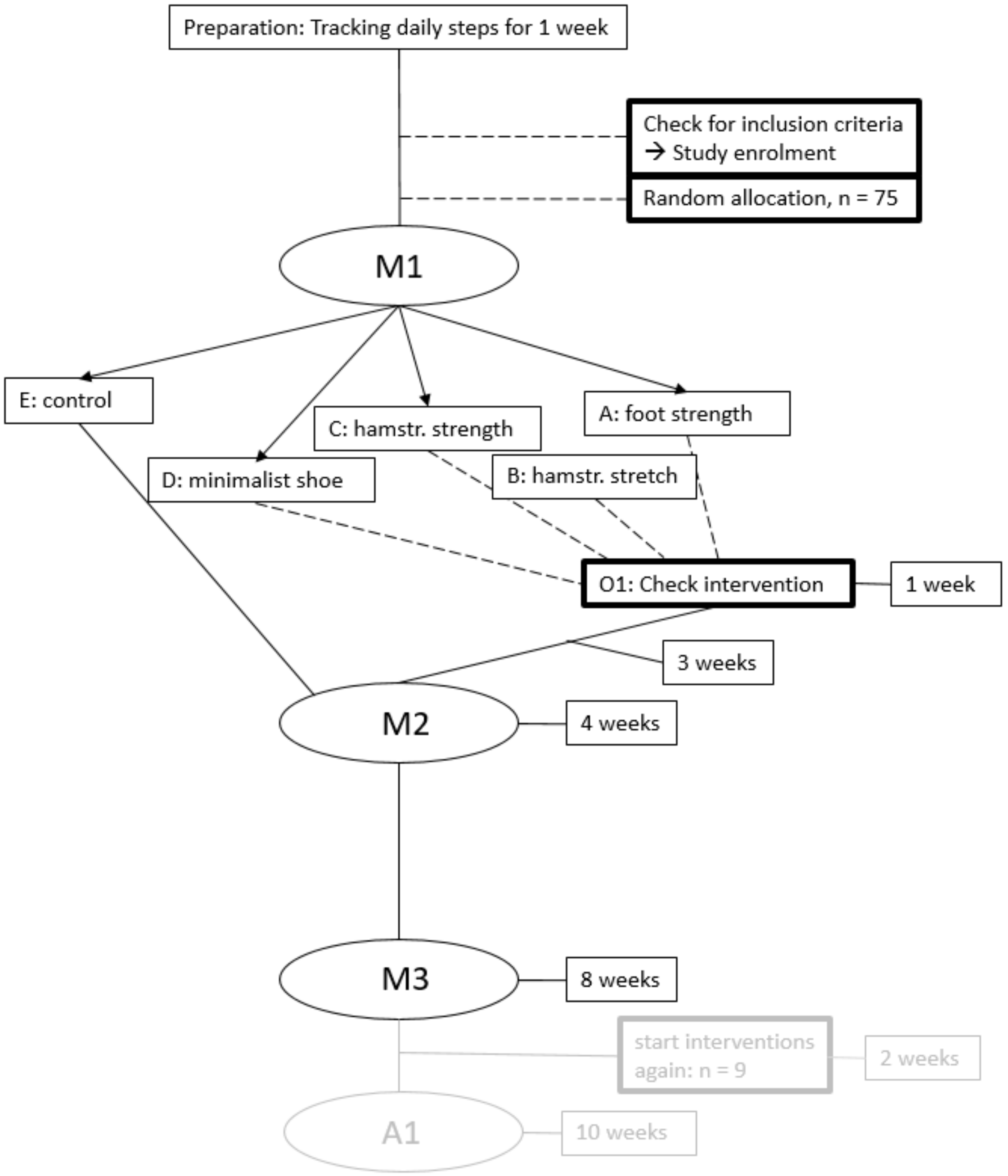

Figure 1. Graphic representation of study procedure; Note: M1, on-site measurement 1 (baseline); M2, on-site measurement 2 (follow-up 1); M3, on-site measurement 3 (follow-up 2); A1, additional on-site measurement (follow-up 3 after wash-out phase)

7. Study population

a. Inclusion and exclusion criteria

Inclusion:

- healthy participants (m/w/d)
- aged 18-40 years
- Recreationally active
    - which is defined as "participating in at least 20-60 minutes of moderate to vigorous intensity physical activity 3-5days/week". Moderate intensity is typically defined as 3-6 METS (e.g. brisk walking) whereas vigorous is activities over 6 METS (jogging or running) (Beierle et al., 2019)
    - and/including walking at least around 5000-10000 steps a day (measured in the week before)(Tudor-Locke et al., 2011)

Exclusion:

- no consent to participate
- regular barefoot runners/walkers
- experienced with short foot/toe grip exercises = intrinsic foot muscles exercises
- professional athletes
- pregnancy
- diseases: diabetes mellitus/CRPS; childhood foot deformities; diagnosed flatfoot; pain in elbow, shoulders or foot; musculoskeletal injury or in the lower extremity in the last 12 months (incl. foot); history of surgery in the lower limb and foot; self-reported disability due to neurological/musculoskeletal impairment in the lower extremity, that makes participants unable to plantar-flex the toes e.g. or would affect motor function in general; osteoarthritis affecting the foot; plantar fasciitis; shin splits; any history of ankle or foot sprain, fracture in the leg or foot; neurological or vestibular impairment that affected balance; lumbosacral radiculopathy; soft tissue disorder such as Marfan or Ehlers-Danlos syndrome, vascular disorders that affect blood circulation in the lower extremity, skin disease in the foot area.

b. Number of study participants

n=15 per group (total n=75)

c. Means of recruitment

- University setting (students and employees)
- University recruitment system
- Online social media advertisement in specific groups (e.g. Studienstiftung)
- Flyer

8. Order of study

When participants are planned to be enrolled in the study (prior to the first on-site session M1), the inclusion criteria will be checked via a checklist/questionnaire. Further, they will be asked to report the average amount of steps per day (recommend to be tracked with a pedometer App (Accupedo, Corusen, US). via smartphone or smartwatch for one week) and the leg dominance (leg preferred for kicking a ball). If they fulfil all the inclusion criteria (also steps/day), participants are enrolled in the study. They will receive further study information (including timeline, measurement devices and

measurement procedure etc.) via mail and self-recorded video then. Participants are asked to maintain their regular levels of activity and not introduce any new fitness training routines during the study (Mulligan & Cook, 2013).

Then, we randomly allocate them to one of the five groups. It must be considered that due to practical reasons, the measurements will be performed in two blocks. In the first block, we will start the measurements with groups A, B and E only. Therefore, group randomisation will be performed between these three groups in the beginning, only. After the measurements for intervention A and B and the control group are finished, we will continue participant recruitment for interventions C and D. For the second measurement block, randomisation will be performed between group C and D. Therefore, we will perform two computer generated sequence random allocations for the measurement block 1 (A, B, E) and 2 (C, D) (Figure 2).

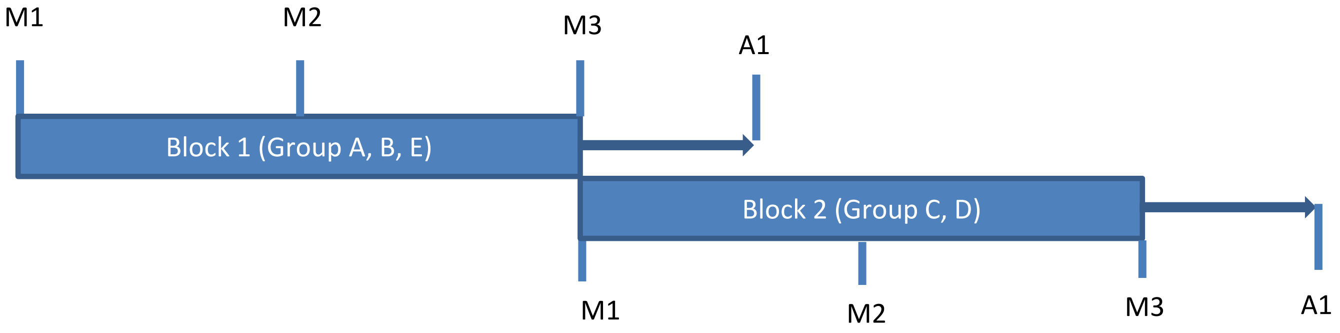

Figure 2. Measurements will be performed in two blocks, where the intervention A,B and control (E) are included in block 1 and intervention C and D in block 2

The series of random allocation will be created using a web application (https://www.random.org accessed on 22 November 2021), and is revealed to the examiner but not to the examinees, who are blinded to the potential effects of the treatment protocols (Fonta, Tsepis, Fousekis, & Mandalidis, 2021). The online sequence generator will randomize an integer sequence (number of participants). The list of random participant numbers is then divided into the respective groups.

Participants will attend three on-site sessions (all approximately 1 hour) in the laboratory of the faculty of sports- and health sciences (TUM). Participants are instructed to avoid both, strenuous physical activity and excessive consumption of alcohol or stimulants, such as caffeine, in the 24 h prior to the test sessions.

In the **first in-person session (M1)**, participants sign the informed consent. Then, the baseline measurement is performed. Following that, participants are instructed concerning the intervention or control procedure.

Then, between baseline and post-measurement, participants perform **four weeks of intervention (or no intervention)** with one **online monitoring session after one week (O1)** for all groups. **After four weeks of intervention** (or no intervention), the first **post-measurement (M2)** is conducted at the same daytime as the baseline measurement.

There will be a third measurement **(M3)**, nine weeks after the baseline measurement and five weeks after the second measurement and the end of the intervention/control, respectively (Taddei et al., 2018).

We also plan to investigate the outcome parameter in a few participants of our sample after this wash-out phase (A1). After the third measurement (M3), they will start to perform their intervention, again, for two weeks. Then, they will be measured again. This would allow us to investigate, whether the changes –if there are any- caused by the interventions could be regained within a shorter period of time, after the wash out –if there is any-.

a. Methods of Study information and obtaining consent

Participants will be informed about the study procedure and conduct in the written invitation. Further questions are answered (via mail and if requested via phone) and then participants are asked to provide written informed consent on the first in-person session (M1). Still, the participants do not receive further information concerning the intervention methods and our expected outcomes a priori. They will get more specific information on the respective intervention according to the group allocation at M1.

b. Intervention / Control

There will be four interventions: A: foot strength training, B: hamstrings stretching, C: hamstrings strength training, D: minimalist shoe walking and one control group (E). All interventions, except D, will be performed with the dominant leg.

The intervention A, B and C are performed barefoot (Fraser & Hertel, 2019; Sulowska et al., 2019). In the beginning (M1), the main investigator gives verbal instructions and provides a demonstration of the exercises, so that participants are familiarized with the exercises and they get additional written instructions as short information and "reminder" (Jung et al., 2011; Sulowska et al., 2019). In addition, we use a web-based training software (Lanista, MP Sports, Coaching & Consulting GmbH, GE), which contains self-recorded explanation videos with walkthroughs and descriptions of each exercise of the protocol, which could be accessed only by a login, which participants receive via mail after the first measurement (M1) (Taddei et al., 2018). The software is also used as log diary, where participants record if they performed the daily exercises and to which amount (see also 11. data protection) (Fraser & Hertel, 2019; Mulligan & Cook, 2013; Taddei et al., 2018). Participants are recommended to e.g. set an alarm clock or fix a specific daytime to be remembered and do not forget to perform the exercises. The interventions will be performed daily for 4 weeks and there will be one online supervised session after one week (O1), in which the physiotherapist verifies if the performance of each exercise is executed according to the protocol, providing help and tips if necessary (Taddei et al., 2018).

A: foot strength training

For this intervention, participants will be instructed to sit on a chair and bend the hip joint, knee joints, and ankle joints to 90°.

In current literature, the duration of the foot strength intervention varies between 4 weeks (Fraser & Hertel, 2019; Mulligan & Cook, 2013), 5 weeks (Kim & Kim, 2016; Lynn, Padilla, & Tsang, 2012), 6 weeks (Sulowska et al., 2019; Unver, Erdem, & Akbas, 2019), 7 weeks (Goldmann, Sanno, Willwacher, Heinrich, & Brüggemann, 2013) and 8 weeks (Taddei et al., 2018). The training frequency in studies varies between 7 days (2 under supervision, 5 home-based) (Unver et al., 2019), (1 under supervision) (Fraser & Hertel, 2019; Sulowska et al., 2019) (unsupervised) (Lynn et al., 2012; Mulligan & Cook, 2013) and four days (Goldmann et al., 2013).

Some studies report that the difficulty of the exercises was progressed after one (Fraser & Hertel, 2019) or two weeks (Lynn et al., 2012; Sulowska et al., 2019; Unver et al., 2019) or when subjects felt that they can perform the exercise properly without great exertion (Fraser & Hertel, 2019; Mulligan & Cook, 2013). The

progression mostly includes changing the exercise position from sitting to standing and/or including different training tools (Fraser & Hertel, 2019; Jung et al., 2011; Lynn et al., 2012; Mulligan & Cook, 2013; Sulowska et al., 2019; Unver et al., 2019).

As we do not intend to perform the exercise in a standing position in order to guarantee that only the foot muscles are exercised, we decided that the exercises should be progressed in a sitting position with a Thera-Band. Fraser and Hertel (2019) suggest that during a four week program there is none to little progression in motor performance expected. Nevertheless, we expect improvements in coordination and therefore, after two weeks, similar to intervention C, the intensity of the exercises (2,3 and 5) is progressed.
The motor training session should be performed in two bouts with each lasting approximately 6 minutes (as B and C). Session 1 includes the warm-up (1) and the hallux-extension- (2) and lesser-toe-extension exercise. Session 2 includes the warm-up (1) and the toe curl (4) as well as the short foot exercise (5).

(1) The toe-spread-out exercise is performed as a warm-up by sequentially by extending all the toes, followed by simultaneously abducting all 5 toes. Then the first and fifth toes are flexed to the ground while the toes 2-4 were kept extended. After that, also the middle toes are relaxed (Fraser & Hertel, 2019; Gooding, Feger, Hart, & Hertel, 2016; Taddei et al., 2018). Participants are instructed to repeat the procedure for 10 times as a warm-up and to increase focus on the foot.

(2) The hallux-extension exercise is performed by extending the first metatarsophalangeal joint while maintaining/pressing the lesser toes (2-5) in contact with/to the floor (Fraser & Hertel, 2019; Gooding et al., 2016; Taddei et al., 2018). The position is held for 5 seconds and repeated for 10 times and 3 sets (30 repetitions) with a 10 s pause after each set (Mulligan & Cook, 2013). After two weeks, participants are instructed to place a (folded) Thera-Band (red) under the lesser toes (2-5) during the exercise. They should try to remove the band with their hands, which means that the intensity with which they press the toes to the ground is increased.

(3) The lesser-toe-extension exercise is performed by extending toes 2-5, while maintaining/pressing the hallux in contact with/to the ground (Fraser & Hertel, 2019; Gooding et al., 2016; Taddei et al., 2018). The position is held for 5 seconds and repeated for 10 times and 3 sets (30 repetitions) with a 10 s pause after each set (Mulligan & Cook, 2013). After two weeks, participants are instructed to place a (folded) Thera-Band (red) under the hallux during the exercise. They should try to remove the band with their hands, which means that the intensity with which they press the toes to the ground is increased.

(4) For the toe curl exercise, a towel is placed under the foot on a slick surface (tile, hardwood floor, etc). Participants are instructed to place their toes on the edge of the towel. They are then instructed to slowly drag the towel under their foot with interphalangeal (IP) and metatarsophalangeal (MTP) flexion of their toes, generating a maximum grip on the fabric for 5 seconds per repetition. The position is held for 5 seconds and repeated for 10 times and 3 sets (30 repetitions) with a 10 s pause after each set (Jung et al., 2011; Lynn et al., 2012; Taddei et al., 2018).

(5) The short-foot exercise is performed by shortening and raising the medial longitudinal arch by actively bringing the first metatarsal head toward the calcaneus in an anteroposterior direction without flexing the toes or contracting the extrinsic foot muscles and then – in shortened position, balance loading of the three support points of the foot = actively press onto ground (heel, metatarsal head 1 and 5). During the exercises, the feet are kept on the ground and the toes are relaxed and not flexed (Gooding et al., 2016; Jung et al., 2011; Kim & Kim, 2016; Sulowska et al.,

2019; Taddei et al., 2018; Unver et al., 2019). The position is held for 5 seconds and repeated for 10 times and 3 sets (30 repetitions) with a 10 s pause after each set (Lynn et al., 2012; Unver et al., 2019). After two weeks, participants are instructed to place a (folded) Thera-Band (red) under the first metatarsal head during the exercise. They should try to remove the band with their hands, which means that the intensity with which they press the joint to the ground is increased.

B: hamstrings stretching exercise

Participants are instructed to perform the stretching program on the dominant side for 4 weeks. They are asked to perform two hamstrings stretching exercises for a total of 6 minutes for two times per day, because previous studies demonstrated that the stiffness of the hamstrings decreased after 5 minutes of stretching (Nakao et al., 2021). For the treatment of plantar fasciitis, it is also recommended to perform a plantar-specific stretch more than once a day (Rathleff et al., 2015). Yahata et al. (2021) investigated the effect of a 60min/week stretching program for 5 weeks, which is according to them 2-to13 times greater to what have been tested in literature. Therefore, -assuming that participants might not perform the exercise every day and not always to the full amount of time- the intensity of the intervention time wise seems to be high enough.

For both exercises (see below a and b), participants are asked to hold a position of mild discomfort, defined as an intensity of 6-7 out of 10 on a numeric rating scale are targeted. For both exercises, participants are instructed **not to flex the spine and to maintain a neutral ankle position** without activation of the M.tibialis anterior to not induce tension on the plantar surface. Participants will be asked to perform three one-minute bouts for each exercise with a ten-second pause between each one-minute bout of stretching.

In various recent stretching studies unilateral stretching protocols for ≥4 weeks were used and no side effects or related injuries were documented (Nakamura et al., 2021; Panidi et al., 2021; Yahata et al., 2021). Due to the increase in flexibility in the contralateral leg to a similar extend like in the stretched leg (Nakamura et al., 2021; Nakamura et al., 2022; Panidi et al., 2021; Yahata et al., 2021) unilateral stretching can be considered as safe.

The first exercise (a) (Figure 3) is carried out standing. The heel of the dominant leg is placed on a table and the other leg is straightened (Wilke et al., 2017). As the participant can increase the intensity of the exercise with leaning the torso forward, the height of the table must not be standardized necessarily.

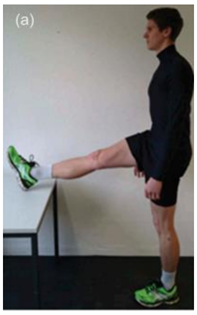

Figure 3. Stretching exercise (a) as described by Wilke et al., 2017

For the second exercise, participants perform a classical sit-and-reach exercise with only the dominant leg (b). This is similar to the position chosen for the back saver sit-and-reach test (Hui & Yuen, 2000; López-Miñarro, de Baranda Andújar, & RodrÑGuez-GarcÑa, 2009). As for exercise a, participants can increase intensity by leaning forward.

C: hamstrings strengthening exercise

As for the stretching exercise, for the hamstrings strengthening intervention, two exercises will be performed in two different positions with a Thera-Band for 2 times (each 6 minutes) per day. With the chosen positions we try to prevent from potential cross-over effects (e.g. single-leg bridging). Exercise (a) will be performed in prone position (Figure 4). The second exercise (b) will be performed in a sitting position (Figure 5). Participants are instructed to perform the exercises with a focus on the concentric movement (2-0-1) for 3x10 repetitions each exercise followed by a 30s pause after each of the three sets. This results in a total loading time of 1.5 minutes and a total pause time of 1.5 minutes (=3 minutes) per exercise (2 exercises for 3 minutes = 6 minutes).
Further, participants are instructed to choose the intensity of the exercise/tension of the Thera-Band so that the last repetition is “just possible”, meaning that they their muscle should feel exerted after the two exercises. After two weeks, participants should switch from the green Thera-Band to the black one.

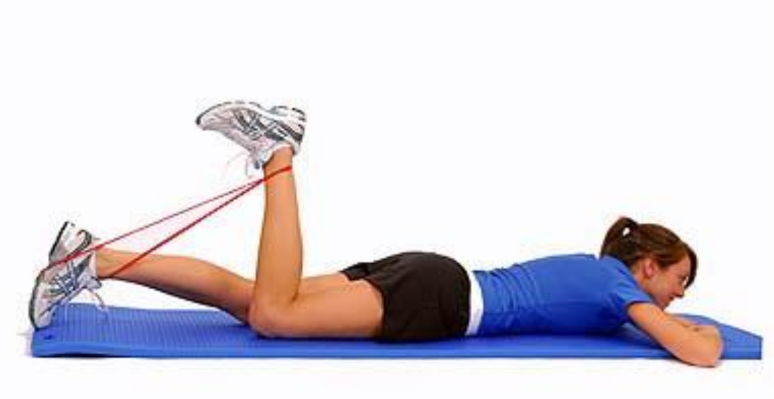

Figure 4. Single leg hamstrings curl (a) with elastic band in prone position © www.performancehealthacademy.com

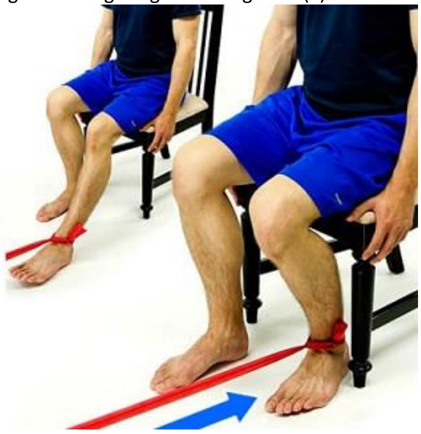

Figure 5. Single leg hamstrings curl (b) with elastic band in sitting position © https://www.knee-pain-explained.com/hamstring-strengthening-exercises.html

D: Minimalist shoe walking

According to Tudor-Locke et al. (2011) adults aged 20-65 years talking less than 5000 steps are classified as sedentary. Therefore, we only include participants talking 5000 or more steps a day (5000-7499 low active, 7500-9999 somewhat active). Participants should start with walking around a third of their daily steps (around 2500-3000) in their minimalist shoes in the first week (Leguano, leguano GmbH, GE). If they are doing fine and there are no adverse events or negative side effects at the time point of the online control appointment (O1), participants are asked to increase their daily steps in

minimalist shoes up to around 5000 after O1 (Sarah T Ridge et al., 2018). The amount of steps is recorded with a pedometer App (Accupedo, Corusen, US) and noted in the online diary log of the participants.

E: control group
The participants of the control group are instructed to continue their usual (sports) routine and not to modify their usual physical activity level. In addition, they also should track usual daily sports routine in the online tool.

c. Acquisition of Outcomes

For investigating our outcomes of interest, several measurement methods would be suitable. The table below shows an overview. We decided for the respective measurement method written in bold letters. The decision was based either on superior validity, reliability or practicability.

Table 1. Summary of suitable measurement methods for the outcome parameters of interest. The measurement methods in bold letters will be applied in our study.

**ROM DC**	SR	Modified SR	Backsaver SR	**Modified backsaver SR**
**ROM ankle/toes**	**Knee to wall test**	**Loaded lunge MT1 ROM**		
**Performance DC**	**Bunkie Test**	**isokinetic measurement (only hamstrings)**	**Standing Isometric DC test**	
**Foot strength direct**	dynamometer	paper grip test	**Pressure platform (RS scan)**	
**Foot strength indirect**	FMM	ND	**ARI (AHI)**	**Modified SEB and CoP**
**Foot position**	**FPI-6**	MLAA	RFA	TA/MLA+foot length

Note; ROM: range of motion; DC: dorsal chain; SR: sit-and-reach test; FMM: foot mobility measurement; ND: navicular drop; ARI: arch rigidity index; AHI: arch height index; FPI-6: foot posture index; MLAA: medial longitudinal arch angle; RFA: rearfoot angle

Participants will be familiarized with the testing procedures via a self-recorded familiarization video. For every participant, the same time-of-day will be chosen for pre-and post-measurements (Guariglia et al., 2011). In the beginning, leg dominance is determined as the ‘preferred leg for kicking a ball’. For every measurement where both legs are tested separately, in order to control for learning effects, the first leg to be tested, will be subsequently changed between the dominant and the non-dominant leg within each group. This allocation will then be applied for all tests, in which one leg is tested after the other and for all 3 measurements. Meaning the first participant of group A will get the dominant leg tested first and the non-dominant second and for the second participant of group A, the non-dominant leg will be tested first.

The measurements (and warm-up) will be performed in the following order:

Warm-up → foot position → foot strength indirect → ROM foot/toes → ROM DC → foot strength direct → performance DC

(1) Warm-up

Subjects perform 5 minutes of cycling at 80W for a warm-up.

(2) The Foot Posture Index (FPI-6), transversal arch and total foot length

The Foot Posture Index (FPI-6) includes six parts which evaluate particular components of the forefoot and the rearfoot in the three cardinal body planes: (1) Talar head palpation, (2) Supra and infra lateral malleolar curvature, (3) Inversion/eversion of the calcaneus, (4) Prominence in the region of the talonavicular joint, (5) Height and congruence of the medial longitudinal arch (6) Abduction/adduction of the forefoot on the rearfoot. The scoring system uses a 5-point Likert-type scale where lower scores represent a more supinated foot position and higher scores a more pronated position. Each of these parts is rated on a scale from -2 to +2. The neutral position of the foot was categorized as 0.The total FPI-6 result classified the foot into the following categories: (i) from -12 to -5: increased foot supination, (ii) from -4 to -1: slight foot supination, (iii) from 0 to +5: neutral foot, (iv) from +6 to +9: slight foot pronation, (v) from +10 to +12: increased foot pronation. The FPI-6 shows good internal construct validity including good individual item fit and good overall fit to the model. The FPI-6 raw scores can be converted to Rasch transformed scores, thereby allowing data generated to be used as interval data (Keenan, Redmond, Horton, Conaghan, & Tennant, 2007). The FPI has proven adequately reliable in varied clinical settings (Intraclass correlation coefficients = 0.62–0.91) and adequate concurrent validity when compared to a skin-mounted-sensor defined static model (Redmond, Crosbie, & Ouvrier, 2006) (Unver, 2019). It is the best clinical measure in terms of reliability compared to commonly used assessments like the navicular drop test (Langley, Cramp, & Morrison, 2016).

The user guide and manual is available online:
https://skiersynergy.com/images/pages/Docs/documents/The%20Foot%20Posture%20Index.pdf

Participants are asked to stand on the pressure plate (RS Scan) with the weight equally distributed between legs, heel and forefoot. Then, foot position data is assessed statically. After the measurement, the width of the transversal arch, the total foot length and the length of the medial longitudinal arch are measured with the software.

(3) Arch rigidity index (ARI)

The arch rigidity index (ARI) is based on the arch height index. The ARI is calculated by dividing the standing arch height index by the sitting arch height index and it represents the structural mobility of the medial longitudinal arch (Mulligan & Cook, 2013). The arch height index is calculated by dividing the height of the dorsum of the foot by the truncated length of the foot to obtain a ratio in both seated and standing positions (Tourillon et al., 2019). An ARI close to 1 represents a stiffer arch while increasing foot flexibility correlates with numbers that rise well above 1. Usually, the ARI measurement

is performed with an arch height index measurement system, which we reconstruct as reported by Mulligan and Cook (2013).

Participants are sitting in a chair with the hips and knees flexed to 90° and maintain a subtalar joint neutral position (STJN). The foot is placed on a sheet of paper, fixed to the floor. The subtalar joint neutral (STJN) position is identified through palpation of the medial and lateral talar head while the participants pronate and supinate their STJ by elevating and collapsing their arch. The STJN position is defined as talonavicular congruency and manually determined by the position in which the talar head depth is symmetrically palpable on both sides. Before the further measurements, similar to the standardized arch height index measurement system, the most posterior calcaneal point of the foot is marked with a weight, fixed to the ground.

Using a cloth tape measure, the **total and truncated lengths of the foot** are measured in a seated position with approximately 10% of the body weight on the foot. The total foot length is the distance from the most posterior aspect of the calcaneus to the tip of the longest toe. The truncated length is the distance from the most posterior aspect of the calcaneus to the center of the first metatarsal head. The position at 50% of the total foot length is marked with a pen.

Then, the dorsum foot height is measured at 50% of the total foot length with a caliper (modified carpenters square). After both feet are measured, subjects are asked to stand up in a relaxed position, where no attempt will be made from the examiner that participants maintain a STJN position. Participants should bring around 90° of the body weight to the measured foot and are allowed to hold on to a wall (only with the fingers) on the contralateral side in order to be able to stand still. The measurement procedure is then repeated. For the standing and the sitting position, different colors are used for marking on the sheet of paper.

(4) Range-of-motion first metatarsophalangeal joint and ankle

For assessing ankle range of motion, participants will perform the knee to wall test, a weight-bearing lunge with the great toe 10 cm away from a wall. A standard tape measure is placed on the floor and participants are instructed to place their foot in a way, the big toe and the heel are aligned with the tape. Participants are allowed to hold on to the wall and instructed to bend the knee (in line with the second toe) in order to touch the wall. If participants are able to touch the wall from that distance. The foot is progressed away from the wall 1 cm at a time and the participant repeat the lunge until they are unable to touch the wall with their knee without lifting the heel from the ground. Once the knee is not able to touch the wall, the foot is progressed in smaller increments toward the wall until the knee makes contact with the wall with the heel in contact with the ground. The test is a reliable measure for assessing ankle range of motion (Powden, Hoch, & Hoch, 2015).

For the range of motion assessment of the first metatarsophalangeal joint, participants also perform a lunge position and are allowed to hold on to the wall. Now the posterior leg/toe is assessed. Participants are instructed to lift the dorsal heel until the first metatarsophalangeal joint lifts off the ground. They are allowed to flex the anterior and posterior knee as much as they want. The movement is repeated for three times. Then, the maximum range of motion (metatarsophalangeal joint still touches the ground) value is measured with a goniometer.

(5) Sit-and-Reach test (modified version)

The back saver sit-and-reach test is an alternative to the classical sit-and-reach test, which is commonly applied to assess flexibility of the hamstring and lower back. The back saver sit-and reach test allows to test one leg at a time and additionally considers leg length discrepancies (López-Miñarro et al.,

2009). Nevertheless, there can occur discomfort in the contralateral hip joint and its validity is not that good in men. Therefore, the modified back saver sit-and-reach test is recommended. Its validity in measuring hamstrings flexibility is moderate (Hui & Yuen, 2000). For the test, the participant sits on a bench with a height of approximately 30cm. The untested leg is placed on the floor with knee flexed approximately 90°. The advantages of this test versions are that only one leg is tested at a time so as to allow evaluation of each leg flexibility separately and that the uncomfortable feeling of the untested leg is minimized compared to the back saver sit-and-reach test. In the original modified back saver sit-and-reach test it is said that there is no sit-and-reach box required. Nevertheless, in order to improve standardization of the foot position, the measurement procedure and evaluation, we will place the classical sit-and-reach box on the bench and participants will perform the test with the box. As described in prior studies, three trials will be performed per leg and the best score will be used for further analysis.

(6) Dynamic balance: Modified star excursion balance test (mSEBT) or COP

For performing the mSEBT, the order of the tested legs will be chosen according to the randomization for all measurements. The direction will be randomized for each testing session. Before testing, the leg length of each participant is evaluated for both legs by measuring the distance from the trochanter major to the floor (barefoot) with a measuring tape. First, participants will complete three practice trials (Robinson) in each direction on each leg. They are instructed to keep the supporting leg in the given position (middle of the “Y”) and not to lift the heels of foot off the ground. They should place the hands on the pelvis (crista iliaca) and keep the hands in that position during the movement. With the second leg they should try to reach as far as they can in the anterior, posterior-medial and posterior-lateral direction, predefined and displayed by the tape and “tip” the maximum point with their toes. Second, to control for fatiguing effects, participants will rest for two minutes. Following that, participants perform 3 test trials in each direction for each leg. A trial is considered as invalid if the participant removes the hands from the hips, does not return to the starting position, lifts or moves the supporting foot during the test or put weight on the moving leg/toes instead of only “tip” in order to achieve greater reach. If there is an invalid trial, the trial is repeated. The maximized reach distance (%MAXD) is calculated using the formula (excursion distance/limb length) x100 = %MAXD (Robbins, Caplan, Aponte, & St-Onge, 2017; Robinson & Gribble, 2008; Ruhe, Fejer, & Walker, 2010).

In addition, the centre-of-pressure (CoP) is evaluated using the pressure platform (RSScan, Logemas, AUS). Therefore, participants are instructed to perform a single-leg stance on the platform and close the eyes. The following ten seconds are recorded. Then, the procedure is repeated with the second leg (Kim & Kim, 2016; Lynn et al., 2012).

(7) Foot strength evaluation using pressure platform

Foot isometric strength is measured using a pressure platform (RSScan, foot scan systems, Logemas, AUS). Participants place one foot on the platform and are instructed to push down on the platform as hard as possible with their hallux first and then with the lesser toes. Maximum force will be normalized by body weight and analyzed for hallux and toes areas separately (Matias, Taddei, Duarte, & Sacco, 2016; Soysa, Hiller, Refshauge, & Burns, 2012; Taddei et al., 2018). The test-retest reliability is excelelnt (Soysa et al., 2012). Further, the doming exercise (=raised medial longitudinal arch) will be performed and analyzed. The movement is instructed as “focus on pulling the ball of the foot towards the heel without curling the toes or raising the ball of the foot off of the ground” (Sarah Trager Ridge, Myrer, Olsen, Jurgensmeier, & Johnson, 2017).

(8) Bunkie Test

The Bunkie Test was conducted as a standardized version of the test described in the original protocol (De Witt & Venter, 2009). We validated the standardized test version in our previous projects (currently under review: Gabriel, Paternoster, Konrad, Horstmann, Pohl; Journal of Sport Biomechanics). Subjects placed their forearms in supine position on a mat with the shoulders over the elbows and the heels on a box (30 cm), both legs straightened. To assess one leg, subjects lifted the pelvis to a neutral position and then raised the contralateral leg about 10 cm off the box. In order to reduce the influence of individual expertise of the investigator on the visual detection of deviations from the neutral position, the horizontal pelvis position was marked with a rubber band, stretched between two fixed stators. Hence, the participants aimed to be in contact with the rubber band throughout the test. In addition, the height of the lifted, contralateral foot was marked with a box (10 cm) (Figure 6). Performance, indicated by the duration of the correctly maintained test position (s), was counted with a stopwatch. The test was stopped if subjects reported any feeling of burning, cramping, pain or strain, ended the test due to fatigue or reached the cut-off score (40 s) according to the original test report (De Witt & Venter, 2009). If subjects were not able to maintain a neutral position, they were verbally corrected by the examiner and allowed to correct the position once. If they were any further deviations, the test was ended. After a 30-s pause, the procedure was repeated for the contralateral leg (Brumitt, 2015; De Witt & Venter, 2009; Ronai, 2015; Van Pletzen & Venter, 2012).

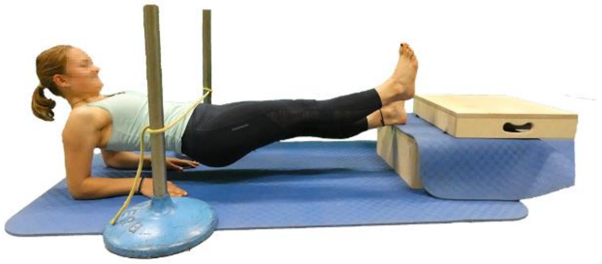

Figure 6. The standardized version of the Bunkie Test.

(9) Standing isometric posterior chain strength test

We will conduct the test as described in the protocol by Matinlauri et al. (2019).

For the isometric posterior chain strength test, participants stand with their leg and lower back against a wall bar. The tested leg is placed with the ankle in a neutral position on a force plate (Kistler 9286BA, Kistler Group, Winterhur, Switzerland), which is placed on a box. The height of the box and its distance to the wall are individually adjusted, so that subjects are tested in 90° hip flexion and 20° knee flexion position. The angles are checked with a goniometer. In the test description by Matinlauri et al. (2019) a researcher fixed the knee of the standing leg to ensure full extension. We adapt that to a strap, passively fixing the participants knee. The researcher carefully will check and correct for additional movements during the test, as for

example rotation of the lumbo-pelvic region. Participants place their hand on the opposite acromion (hand on chest).

The tester gives the instruction; "to exert maximal force vertically into the force plate" and continues verbal motivation for 3.5 s. Each limb is tested two times with 60 seconds pause between. The assessment of both legs is separated by 30 seconds. The force plate sampling frequency is set at 500 Hz with no filter/ smoothing applied. Raw data is exported to a customized spreadsheet and the highest isometric force (IPF) produced in each limb and condition is extracted from each of the trials. The average of the IPF of each limb is used in the analysis.

(10) Isokinetic measurement of the hamstrings

The muscle strength of the knee flexors during concentric movement, can precisely be evaluated via isokinetic testing (ISOMED 2000, D & R Ferstl GmbH, Hemau, Germany). It is calculated as knee flexors peak torque, which we measure dynamically with a range of motion of 85° between 5° to 90° knee flexion. Participants are positioned at the seat and straps are placed over the shoulders, across the waist and over the middle of the thigh to ensure a stable torso and thigh. The axis of rotation of the dynamometer arm is positioned just lateral to the femoral epicondyle.

Subjects get detailed information on the measurement procedure in advance. The test is performed for 5 repetitions at 60°/s and 5 at 120°/s with an one-minute pause in between the two angular speeds. Before each measurement trial, participants perform a test trial with 5 repetitions at sub-maximal force production in order to get familiarized with the movement and the movement speed. The testing procedure is repeated for the respective other leg then (Emrani et al., 2006; Grygorowicz, Kubacki, Pilis, Gieremek, & Rzepka, 2010; Jeon, Seo, & Lee, 2016; Rosene, Fogarty, & Mahaffey, 2001).

Angle and torque values are captured with the measurement software proEMG (prophysics AG, CH). Data is further processed with Matlab (R2020b, MathWorks, US). We calculate the peak torque values of each 5 movements for each angular speed and each leg. Then the average peak torque of the 5 movements for each angular speed and each leg is evaluated thereof.

d. Temporal Sequence and duration of the study for the individual participant

Before the study, participants are asked to track their daily amount of steps for one week (Accupedo, Corusen, US). The inclusion criteria are checked. If participants fulfil the criteria of study participation, they will be familiarized with the measurement and study procedure and measurement devices via e-mail and video. The participants will have to visit the laboratory in-person for 3 measurement session (M1-3), each lasting about 1 hour. During the first in-person session (M1), participants sign the informed consent. We try to reduce the burden of traveling to the laboratory, by switching the

"monitoring-session" after one week intervention to an online format (O1). Further, participants should stick to the respective intervention exercises for 4 weeks and document their amount of exercise online. A few participants of each group are asked to (voluntarily!) be measured again, a fourth time, after 3 weeks (2 weeks wash-out, 2 weeks intervention, again) (A1).

e. Total duration of the study

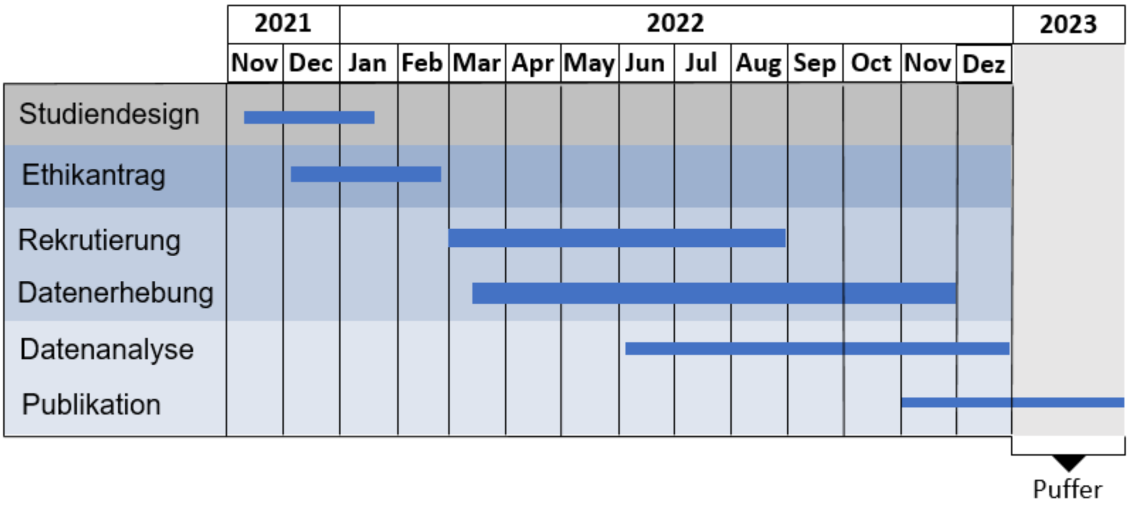

9. Risk-benefit analysis

a. Benefit for the individual participant

Participants could benefit from the applied interventions and measurements. The applied interventions showed health benefits according to prior studies. Further, the participants could get the results and interpretation of their measurement data after the study on request (e.g. isokinetic measurement) for free. In addition, we would like to offer material goods (sports equipment) for compensation.

c. Burden and Risks for the individual participant

The participants will have to visit the laboratory in-person for 3 measurement session (M1-3). We try to reduce the burden of traveling to the laboratory (and reducing in-person time in the laboratory), by providing familiarization with the measurement devices and study procedure via video, allowing to fill out the anthropometric questionnaire online and switching the "monitoring-session" after one week of intervention to an online format. Further, participants should stick to the respective intervention exercises for 4 weeks and document their amount of exercise online. The medical risks are none to minimal as all testing and intervention procedures were already frequently applied in prior studies. Nevertheless, there are some reporting of overuse injury or pain after starting wearing minimalist shoes too much. We try to prevent these, by only recommending the shoes for walking (not running) and starting only with a third of the individually taken steps, to be performed in minimalist shoes. If there are no signs of pain or injury, after one week, participants are encouraged to increase their "barefoot-shoe"-step amount to 5000 steps/day (Accupedo, Corusen, US).

d. Abort criterion

The testing or intervention will be immediately stopped if the participant reports any feeling of extreme discomfort or in the case of unusual events. The subject may without any resulting detriment withdraw from the exploratory study at any time by revoking his or her informed consent. In this case participants' data will be deleted.

e. Statement to medical tenability

All interventions and testing techniques used in this study are non-invasive and commonly used in sports and therapy. Therefore, we expect that our techniques provide none to minimal risk of medical adverse events or unusual negative side effects and medical tenability can be provided.

10. Biometry

Our sample size calculation is based on the effect sizes of prior studies. Cohen´s d was calculated according to the formula:

$$d = \frac{(MN1 - MN2)}{SD_{pooled}}$$

with $SD_{pooled} = \sqrt{\frac{(SD12+SD22)}{2}}$

Where: MN1 = mean of group 1, MN2 = mean of group 2, SD1 = standard deviation of group 1, SD2 = standard deviation of group 2, SDpooled = pooled standard deviation (https://goodcalculators.com/effect-size-calculator/).

For an eight-week program, Taddei et al. (2018) report a large effect size of d=0.997 for the investigation of a foot strengthening program on toe flexor strength. Kim and Kim (2016) compared the effect of short foot exercises and arch support insoles in two groups (n = 7 per group) on foot posture and postural balance. They also report a large effect size of d = 5.54. Fraser and Hertel (2019), who investigated the effect of a 4-week foot strengthening intervention program on motor performance (n = 12 per group, 2 groups), also found a large effect size (d = 1.207) (Fraser & Hertel, 2019).

Also for the investigation of a 4-week hamstrings stretch in 2 groups (n = 15 per group) on hamstrings force, Nakao et al. (2021), report a large effect size of d = 2.145. A further prior study, in which Sulowska et al. (2019) investigated the effect of a four-week foot strengthening program on isokinetic strength in the hamstrings, a large effect size of (d = 3.444) is reported.

Calculating with the large effects size of the eight-weeks program (Taddei et al., 2018) α = 0.05 and β = 0.95 (MANOVA, 5 groups)), the sample size calculation in G*Power (G*Power version 3.1., Heinrich-Heine-University Düsseldorf, Germany) resulted in a sample of n = 15 participants. Taddei et al. (2018) report only a drop-out-rate of 3% for such an intervention

(8-16 weeks). Therefore, our sample seems to be suitable and similar to prior studies e.g. on foot strengthening exercise interventions (n = 12 (Fraser & Hertel, 2019).

a. Confirmatory study

Mixed models of analysis of variance (MANOVA) (5 groups x 3-4times) will be used to detect if groups changed differently over time (treatment-time interactions). Post-hoc repeated measures ANOVA will be performed for each group separately to determine if there are significant changes in the outcome parameters between the measurement points. To determine if any increases are significantly different between groups, the changes in each variable between M1-M2, M2-M3 (and M3-A1) are analysed with ANOVA. The significance level is set at $\alpha = 0.05$.

b. Explorative study

11. Data management and data protection

a. Acquisition, storing (type, place, duration), data transfer, ensurement of data security

Data collection and editing is done for research purpose and all data handling is based on the DS-GVO 2018 (Europäische DatenschutzVO, 2018). We guarantee Data quality according to DIN ISO 9000 (DIN, 2015). Further clinical testing and work of the study will only start if ethical approval is given. All paper-based data, will be safely stored at the professorship of Conservative and Rehabilitative orthopaedics and will be accessible for ten years. Data will only be transferred to an electronical form after anonymisation. The digital form of the data will be stored on a password protected network drive of the professorship for conservative and rehabilitative orthopaedics. Provided that there is no conflict of interest, data can be used after collection and editing from other TUM researchers and guest researchers at TUM. Final study results can be published if demanded. All researchers are under the obligation of documentation, secrecy and restriction to provide data for others – all according to DS-GVO 2018. Participants give their signed informed consent prior to study conduction, in which data handling based on DS-GVO 2018 (Europäische DatenschutzVO, 2018) is stated.

We contacted the person, responsible for data security and protection (TUM), concerning the cooperation with the trainingsoftware Lanista (MP Sports, Coaching & Consulting GmbH, GE) for which we would provide the usage data of the software in an anonymized form. Participants are informed thereof and explicitly asked for consent in the informed consent sheet. They also have the possibility to only choose to participate in the study without allowing the anonymized data of the software usage to be processed to MP Sports, Coaching & Consulting GmbH). We registered a "Verarbeitungstätigkeit" online (https://dsms.datenschutz.tum.de/rpa/pa-details/552).

b. Anonymised / pseudonymised

All measurement data will be anonymised by assigning data to an individual participant code consisting of a letter and sequential numbering before the statistical analyses.

c. Revocation, data deletion

The subject may without any resulting detriment withdraw from the exploratory study at any time by revoking his or her informed consent. In this case participants’ data will be deleted.

12. Handling of biomaterial

Not applicable.

13. Proband insurance

As in our previous studies with similar measurement instruments, for which received a positive vote of the ethical committee (576/20 S-EB and 735/20 S-KH), there is none to little risk of accidents during the testing procedure. Therefore, there will be no insurance for the test persons.

14. Signatures of principle investigator

[redacted]

15. References

Afonso, J., Ramirez-Campillo, R., Moscão, J., Rocha, T., Zacca, R., Martins, A., . . . Clemente, F. M. (2021). *Strength Training versus Stretching for Improving Range of Motion: A Systematic Review and Meta-Analysis.* Paper presented at the Healthcare.

Ajimsha, M., Binsu, D., & Chithra, S. (2014). Effectiveness of myofascial release in the management of plantar heel pain: a randomized controlled trial. *The Foot, 24*(2), 66-71.

Beierle, R., Burton, P., Smith, H., Smith, M., & Ives, S. J. (2019). The effect of barefoot running on EMG activity in the gastrocnemius and tibialis anterior in active college-aged females. *International journal of exercise science, 12*(1), 1110.

Brumitt, J. (2015). The Bunkie test: descriptive data for a novel test of core muscular endurance. *Rehabilitation research and practice, 2015*.

De Witt, B., & Venter, R. (2009). The 'Bunkie'test: assessing functional strength to restore function through fascia manipulation. *Journal of Bodywork and movement therapies, 13*(1), 81-88.

Emrani, A., Bagheri, H., Hadian, M. R., Jabal-Ameli, M., Olyaei, G. R., & Talebian, S. (2006). Isokinetic strength and functional status in knee osteoarthritis. *Journal of physical therapy science, 18*(2), 107-114.

Fonta, M., Tsepis, E., Fousekis, K., & Mandalidis, D. (2021). Acute Effects of Static Self-Stretching Exercises and Foam Roller Self-Massaging on the Trunk Range of Motions and Strength of the Trunk Extensors. *Sports, 9*(12), 159.

Fraser, J. J., & Hertel, J. (2019). Effects of a 4-week intrinsic foot muscle exercise program on motor function: a preliminary randomized control trial. *Journal of sport rehabilitation, 28*(4), 339-349.

Gabriel, A., Konrad, A., Roidl, A., Queisser, J., Schleip, R., Horstmann, T., & Pohl, T. (2021). Myofascial Treatment Techniques on the Plantar Surface Influence Functional Performance in the Dorsal Kinetic Chain. *Journal of Sports Science and Medicine, 21*(1), 13-22.

Goldmann, J.-P., Sanno, M., Willwacher, S., Heinrich, K., & Brüggemann, G.-P. (2013). The potential of toe flexor muscles to enhance performance. *Journal of sports sciences, 31*(4), 424-433.

Gooding, T. M., Feger, M. A., Hart, J. M., & Hertel, J. (2016). Intrinsic foot muscle activation during specific exercises: a T2 time magnetic resonance imaging study. *Journal of athletic training, 51*(8), 644-650.

Grieve, R., Goodwin, F., Alfaki, M., Bourton, A.-J., Jeffries, C., & Scott, H. (2015). The immediate effect of bilateral self myofascial release on the plantar surface of the feet on hamstring and lumbar spine flexibility: A pilot randomised controlled trial. *Journal of Bodywork and movement therapies, 19*(3), 544-552.

Grygorowicz, M., Kubacki, J., Pilis, W., Gieremek, K., & Rzepka, R. (2010). SELECTED ISOKINETIC TESTS IN KNEE INJURY PREVENTION. *Biology of Sport, 27*(1).

Guariglia, D., Pereira, L., Dias, J., Pereira, H., Menacho, M., Silva, D., . . . Cardoso, J. (2011). Time-of-day effect on hip flexibility associated with the modified sit-and-reach test in males. *International journal of sports medicine, 32*(12), 947-952.

Hollander, K., Heidt, C., Van der Zwaard, B. C., Braumann, K.-M., & Zech, A. (2017). Long-term effects of habitual barefoot running and walking: a systematic review. *Med Sci Sports Exerc, 49*(4), 752-762.

Hui, S.-C., & Yuen, P. Y. (2000). Validity of the modified back-saver sit-and-reach test: a comparison with other protocols. *Medicine and science in sports and exercise, 32*(9), 1655-1659.

Jeon, K., Seo, B.-D., & Lee, S.-H. (2016). Comparative study on isokinetic capacity of knee and ankle joints by functional injury. *Journal of physical therapy science, 28*(1), 250-256.

Johnson, A., Myrer, J., Mitchell, U., Hunter, I., & Ridge, S. (2016). The effects of a transition to minimalist shoe running on intrinsic foot muscle size. *International journal of sports medicine, 37*(02), 154-158.

Jung, D.-Y., Kim, M.-H., Koh, E.-K., Kwon, O.-Y., Cynn, H.-S., & Lee, W.-H. (2011). A comparison in the muscle activity of the abductor hallucis and the medial longitudinal arch angle during toe curl and short foot exercises. *Physical Therapy in Sport, 12*(1), 30-35.

Keenan, A.-M., Redmond, A. C., Horton, M., Conaghan, P. G., & Tennant, A. (2007). The Foot Posture Index: Rasch analysis of a novel, foot-specific outcome measure. *Archives of physical medicine and rehabilitation, 88*(1), 88-93.

Kim, E.-K., & Kim, J. S. (2016). The effects of short foot exercises and arch support insoles on improvement in the medial longitudinal arch and dynamic balance of flexible flatfoot patients. *Journal of physical therapy science, 28*(11), 3136-3139.

Langley, B., Cramp, M., & Morrison, S. C. (2016). Clinical measures of static foot posture do not agree. *Journal of foot and ankle research, 9*(1), 1-6.

López-Miñarro, P. A., de Baranda Andújar, P. S., & RodrÑGuez-GarcÑa, P. L. (2009). A comparison of the sit-and-reach test and the back-saver sit-and-reach test in university students. *Journal of Sports Science & Medicine, 8*(1), 116.

Lynn, S. K., Padilla, R. A., & Tsang, K. K. (2012). Differences in static-and dynamic-balance task performance after 4 weeks of intrinsic-foot-muscle training: the short-foot exercise versus the towel-curl exercise. *Journal of sport rehabilitation, 21*(4), 327-333.

Matias, A. B., Taddei, U. T., Duarte, M., & Sacco, I. C. (2016). Protocol for evaluating the effects of a therapeutic foot exercise program on injury incidence, foot functionality and biomechanics in long-distance runners: a randomized controlled trial. *BMC musculoskeletal disorders, 17*(1), 1-11.

Matinlauri, A., Alcaraz, P. E., Freitas, T. T., Mendiguchia, J., Abedin-Maghanaki, A., Castillo, A., . . . Cohen, D. D. (2019). A comparison of the isometric force fatigue-recovery profile in two posterior chain lower limb tests following simulated soccer competition. *PloS one, 14*(5), e0206561.

Mulligan, E. P., & Cook, P. G. (2013). Effect of plantar intrinsic muscle training on medial longitudinal arch morphology and dynamic function. *Manual therapy, 18*(5), 425-430.

Nakamura, M., Yahata, K., Sato, S., Kiyono, R., Yoshida, R., Fukaya, T., . . . Konrad, A. (2021). Training and Detraining Effects Following a Static Stretching Program on Medial Gastrocnemius Passive Properties. *Frontiers in Physiology, 12*, 449.

Nakamura, M., Yoshida, R., Sato, S., Yahata, K., Murakami, Y., Kasahara, K., . . . Konrad, A. (2022). Cross-education effect of 4-week high-or low-intensity static stretching

intervention programs on passive properties of plantar flexors. *Journal of biomechanics*, 110958.

Nakao, S., Ikezoe, T., Nakamura, M., Umegaki, H., Fujita, K., Umehara, J., . . . Ichihashi, N. (2021). Chronic effects of a static stretching program on hamstring strength. *The Journal of Strength & Conditioning Research, 35*(7), 1924-1929.

Panidi, I., Bogdanis, G. C., Terzis, G., Donti, A., Konrad, A., Gaspari, V., & Donti, O. (2021). Muscle architectural and functional adaptations following 12-weeks of stretching in adolescent female athletes. *Frontiers in Physiology, 12*.

Powden, C. J., Hoch, J. M., & Hoch, M. C. (2015). Reliability and minimal detectable change of the weight-bearing lunge test: a systematic review. *Manual therapy, 20*(4), 524-532.

Rathleff, M. S., Mølgaard, C. M., Fredberg, U., Kaalund, S., Andersen, K., Jensen, T., . . . Olesen, J. (2015). High-load strength training improves outcome in patients with plantar fasciitis: A randomized controlled trial with 12-month follow-up. *Scandinavian journal of medicine & science in sports, 25*(3), e292-e300.

Redmond, A. C., Crosbie, J., & Ouvrier, R. A. (2006). Development and validation of a novel rating system for scoring standing foot posture: the Foot Posture Index. *Clinical biomechanics, 21*(1), 89-98.

Ridge, S. T., Myrer, J. W., Olsen, M. T., Jurgensmeier, K., & Johnson, A. W. (2017). Reliability of doming and toe flexion testing to quantify foot muscle strength. *Journal of foot and ankle research, 10*(1), 1-7.

Ridge, S. T., Olsen, M. T., Bruening, D. A., Jurgensmeier, K., Griffin, D., Davis, I. S., & Johnson, A. W. (2018). Walking in minimalist shoes is effective for strengthening foot muscles.

Robbins, S. M., Caplan, R. M., Aponte, D. I., & St-Onge, N. (2017). Test-retest reliability of a balance testing protocol with external perturbations in young healthy adults. *Gait & Posture, 58*, 433-439.

Robinson, R. H., & Gribble, P. A. (2008). Support for a reduction in the number of trials needed for the star excursion balance test. *Archives of physical medicine and rehabilitation, 89*(2), 364-370.

Ronai, P. (2015). The bunkie test. *Strength & Conditioning Journal, 37*(3), 89-92.

Rosene, J. M., Fogarty, T. D., & Mahaffey, B. L. (2001). Isokinetic hamstrings: quadriceps ratios in intercollegiate athletes. *Journal of athletic training, 36*(4), 378.

Ruhe, A., Fejer, R., & Walker, B. (2010). The test–retest reliability of centre of pressure measures in bipedal static task conditions–a systematic review of the literature. *Gait & Posture, 32*(4), 436-445.

Soysa, A., Hiller, C., Refshauge, K., & Burns, J. (2012). Importance and challenges of measuring intrinsic foot muscle strength. *Journal of foot and ankle research, 5*(1), 1-14.

Sulowska, I., Mika, A., Oleksy, Ł., & Stolarczyk, A. (2019). The influence of plantar short foot muscle exercises on the lower extremity muscle strength and power in proximal segments of the kinematic chain in long-distance runners. *BioMed research international, 2019*.

Taddei, U. T., Matias, A. B., Ribeiro, F. I., Inoue, R. S., Bus, S. A., & Sacco, I. C. (2018). Effects of a therapeutic foot exercise program on injury incidence, foot functionality and biomechanics in long-distance runners: Feasibility study for a randomized controlled trial. *Physical Therapy in Sport, 34*, 216-226.

Tourillon, R., Gojanovic, B., & Fourchet, F. (2019). How to evaluate and improve foot strength in athletes: An update. *Frontiers in Sports and Active Living, 1*, 46.

Tudor-Locke, C., Craig, C. L., Brown, W. J., Clemes, S. A., De Cocker, K., Giles-Corti, B., . . . Mutrie, N. (2011). How many steps/day are enough? For adults. *International Journal of Behavioral Nutrition and Physical Activity, 8*(1), 1-17.

Unver, B., Erdem, E. U., & Akbas, E. (2019). Effects of short-foot exercises on foot posture, pain, disability, and plantar pressure in Pes Planus. *Journal of sport rehabilitation, 29*(4), 436-440.

Van Pletzen, D., & Venter, R. E. (2012). The relationship between the bunkie-test and physical performance in rugby union players. *International Journal of Sports Science & Coaching, 7*(3), 543-553.

Wilke, J., Vogt, L., Niederer, D., & Banzer, W. (2017). Is remote stretching based on myofascial chains as effective as local exercise? A randomised-controlled trial. *Journal of sports sciences, 35*(20), 2021-2027.

Yahata, K., Konrad, A., Sato, S., Kiyono, R., Yoshida, R., Fukaya, T., . . . Nakamura, M. (2021). Effects of a high-volume static stretching programme on plantar-flexor muscle strength and architecture. *European Journal of Applied Physiology, 121*(4), 1159-1166.
